# First Year Growth in Relation to Prenatal Exposure to Endocrine Disruptors — A Dutch Prospective Cohort Study

**DOI:** 10.3390/ijerph110707001

**Published:** 2014-07-10

**Authors:** Marijke de Cock, Michiel R. de Boer, Marja Lamoree, Juliette Legler, Margot van de Bor

**Affiliations:** 1 Section Health and Life Sciences, Faculty of Earth and Life Sciences, VU University, De Boelelaan 1085, 1081 HV Amsterdam, The Netherlands; E-Mail: m.vande.bor@vu.nl; 2 Section Health Sciences, Faculty of Earth and Life Sciences, VU University, De Boelelaan 1085, 1081 HV Amsterdam, The Netherlands; E-Mail: m.r.de.boer@vu.nl; 3 Institute for Environmental Studies, Faculty of Earth and Life Sciences, VU University, De Boelelaan 1085, 1081 HV Amsterdam, The Netherlands; E-Mails: marja.lamoree@vu.nl (M.L.); Juliette.legler@vu.nl (J.L.)

**Keywords:** endocrine disruption, prenatal exposure, BMI, head circumference, early childhood

## Abstract

Growth in the first year of life may already be predictive of obesity later in childhood. The objective was to assess the association between prenatal exposure to various endocrine disrupting chemicals (EDCs) and child growth during the first year. Dichloro-diphenyldichloroethylene (DDE), mono(2-ethyl-5-carboxypentyl)phthalate (MECPP), mono(2-ethyl-5-hydroxyhexyl)phthalate (MEHHP), mono(2-ethyl-5-oxohexyl)phthalate (MEOHP), polychlorinated biphenyl-153, perfluorooctanesulfonic acid, and perfluoro-octanoic acid were measured in cord plasma or breast milk. Data on weight, length, and head circumference (HC) until 11 months after birth was obtained from 89 mother-child pairs. Mixed models were composed for each health outcome and exposure in quartiles. For MEOHP, boys in quartile 1 had a higher BMI than higher exposed boys (*p* = 0.029). High DDE exposure was associated with low BMI over time in boys (0.8 kg/m^2^ difference at 11 m). Boys with high MECPP exposure had a greater HC (1.0 cm difference at 11 m) than other boys (*p* = 0.047), as did girls in the second quartile of MEHHP (*p* = 0.018) and DDE (*p* < 0.001) exposure. In conclusion, exposure to phthalates and DDE was associated with BMI as well as with HC during the first year after birth. These results should be interpreted with caution though, due to the limited sample size.

## 1. Introduction

Optimal development and health early in life are key factors for health and wellbeing during childhood and adulthood. It has been hypothesized that adult health and disease have their origin in the prenatal and early postnatal environment, a concept referred to as the Developmental Origins of Health and Disease [1]. This is illustrated by the Dutch Hunger Winter Study, in which exposure to famine during gestation was associated with an increased risk for amongst others coronary heart disease and obesity [2]. These associations were furthermore dependent on the timing of the famine during gestation, as they were only observed for those exposed early in pregnancy.

There are various parameters early in life which are indicators for development later in life. Birth weight, for example, is inversely associated with hypertension [3] and type 2 diabetes [4] in adulthood, and both high [5] and low [6] birth weight are associated with obesity. Rapid growth in infancy, in terms of both weight and height, is considered an independent risk factor for childhood obesity [7,8]. A recent study by Gittner *et al.* showed that children who were obese at the age of five years already had distinct body mass index (BMI) patterns before 12 months of age. Children who had a normal BMI at age five always had a lower BMI from age 6 months and onwards compared to children who were obese at age five years [9]. They furthermore showed that BMI patterns over time differed between male and female children, with female children who were overweight at the age of five gaining weight faster in the first two years of life compared to male overweight children [9]. 

Childhood obesity has also been related to exposure to endocrine disrupting chemicals (EDCs) early in life. Several studies have observed positive associations with BMI and early childhood growth for chemicals such as organochlorine pesticides (e.g., dichlorodiphenyltrichloroethane (DDT), hexachlorobenzene (HCB)) [10,11,12,13] and perfluorinated alkyl acids (PFAAs) [14]. On the other hand several studies have reported no effect [15,16,17,18], as we have described in a recently published review [19]. Furthermore associations seem to differ between males and females, and dose-response relations are not clear. Exposure to these chemicals is widespread. The organochlorine pesticides, such as DDT, of which dichlorodiphenyldichloroethylene (DDE) is the major metabolite, and HCB, have been banned from production [20,21], but remain in the food chain due to their lipophilic and bioaccumulating properties. DDT is furthermore still in use in developing countries for vector control (malaria) [22]. Polychlorinated biphenyls (PCBs), which were mainly applied as dielectric and coolant fluids and which were banned due to their carcinogenic characteristics [23], have also bioaccumulated in the food chain [24]. Exposure to these chemicals is slowly decreasing over time, however it is not certain if low dose exposure implies safe exposure [25]. Not all EDCs are persistent, though exposure to non-persistent EDCs may occur on a continuous basis. Examples of these are phthalates which are used to soften plastics, and perfluorinated alkyl acids (PFAAs), surfactants used in various consumer products. Furthermore, the presence of many chemicals, such as PFAAs, PCBs, organochlorine pesticides, and phthalates, can be detected in 99%–100% of pregnant women [26]. As several of these chemicals have also been detected in e.g., cord blood [12,27] and amniotic fluid [28,29], it is clear that the placenta does not protect the fetus from these exposures [30,31]. EDCs are suspected—as implied by their name—to disrupt hormonal action, and exposure early in life may have long lasting effects [32]. Experimental studies have furthermore shown that they may cause epigenetic changes [33], which implies that effects of these chemicals may potentially be trans-generational.

In light of prevention, it is essential to determine to which extent EDCs affect child growth as early as in the first 12 months after birth. As infants in the Netherlands are eligible for free health monitoring by youth health care organizations, each child is measured and weighed regularly, especially in the first twelve months. The objective of this study was to determine possible associations between markers of prenatal exposure to various EDCs and child growth in the first year of life. The compounds included were DDE, PCB-153, three metabolites of di(2-ethylhexyl) phthalate (DEHP), including mono(2-ethyl-5-carboxypentyl)phthalate (MECPP), mono(2-ethyl-5-hydroxyhexyl)phthalate (MEHHP), and mono(2-ethyl-5-oxohexyl)phthalate (MEOHP), as well as the PFAAs perfluorooctanesulfonic acid (PFOS), and perfluorooctanoic acid (PFOA). 

## 2. Methods

### 2.1. Study Procedures and Subjects

Six midwifery clinics in the area of Zwolle, the Netherlands, participated in the recruitment of pregnant women, which started in January 2011 and finished in January 2013. The Zwolle community is located in the “Salland” area, which is characterized by a relatively low level of urbanization. Women were invited to participate during the first antenatal visit to the midwife (between 10 and 12 weeks of pregnancy) and were considered eligible for participation if they were able to fill out Dutch questionnaires. In total 148 mother-child pairs were included. Twin pregnancies and major congenital anomalies were reasons for exclusion, however no participant was excluded because of these criteria. Signed informed consent was obtained from every participant. Cord blood and breast milk were collected for determination of markers of early life exposure to several EDCs. Youth health care organizations in the area of Zwolle were approached for data on growth during the first year which they collect during scheduled follow-up visits of each child in the Netherlands. About 99.5% of parents in the Netherlands visit youth health care centres with their child during the first year [34]. On average each child is seen six times during this period. Information on parental anthropometry was obtained from the midwives, and questionnaires were administered during pregnancy to collect information on parental health and lifestyle, and previous pregnancies. Fourteen subjects dropped-out before delivery due to lack of time of the mother to participate, and for 45 subjects no exposure data was available, resulting in the inclusion of 89 mother-child pairs for analysis. The study was approved by the medical ethics committee of the VU University medical centre.

### 2.2. First Year Growth

Data on weight, height (supine length), and head circumference were obtained from youth health care organizations. Parents were contacted by youth health care for follow-up visits at 1, 2, 4, 6, 9, and 11 months after birth. Infant BMI was calculated from weight and height (BMI = weight (kg)/height^2^ (m^2^)). 

### 2.3. Chemical Exposure

Umbilical cord blood was collected immediately after birth when the health of mother and child was ascertained. Midwives and nurses were instructed to collect as much blood as possible and to transfer it to EDTA tubes. The blood was delivered to the lab within twelve hours by a courier in case of home delivery or by hospital staff in case of delivery at the hospital. At the lab, cord blood was centrifuged for 10 min at 2000 g after which the plasma layer was transferred to plasma tubes. Plasma was stored at −80 °C.

Breast milk was collected in the second month after birth (mean (SD): 6.3 (2.5) weeks). In total a minimum of 100 mL was collected, spread over five to ten days to minimize the burden to the mothers in case of low milk flow. Mothers were instructed to note the dates on which they collected a sample and to store the milk in the freezer in between sampling days. They were allowed to use a breast pump for collection.

Compounds were analysed in cord plasma. For DDE we also analysed breast milk samples from mothers for whom an insufficient amount of cord blood was available. PFOA and PFOS were analysed by applying isotope dilution and large volume injection using an on-line trapping column coupled to liquid chromatography and triple quadrupole mass spectrometry. The isotope labelled standards ^13^C_4_-PFOA and ^13^C_4_PFOS were obtained from Wellington Laboratories (Guelph, ON, Canada). The breast milk samples were extracted with solid phase extraction using Oasis WAX cartridges (Waters, Milford, MA, USA). For the cord plasma samples, the proteins were precipitated by adding methanol and centrifuging the mixture prior to injection onto the analytical system. 

After drying both the cord plasma and the breast milk samples with Kieselguhr (Supelco, Sigma-Aldrich, St. Louis, MO, USA), the organochlorine pesticide p,p’-DDE, and PCB153 were extracted with a mixture of dichloromethane and hexane. As internal standard, ^13^C_12_-PCB153, obtained from Cambridge Isotope Laboratories (Tewksbury, MA, USA), was used. Cleanup of the extracts was done using sulphuric acid silica columns and for the analysis gas chromatography with mass spectrometric detection in negative chemical ionization mode was used. 

For the analyses of the DEHP metabolites, enzymatic deconjugation was carried out. After addition of the internal standards used for isotope dilution, the breast milk samples were extracted using Oasis MAX (Waters) cartridges, while for the cord plasma samples a simple protein precipitation step using formic acid was applied. The isotope labelled standards ^13^C_4_-MEOHP, ^13^C_4_-MEHHP, ^13^C_4_-MECPP and MEHP‒d_4_ were all obtained from Cambridge Isotope Laboratories. The extracts were analysed after large volume injection using an on-line trapping column coupled to liquid chromatography and triple quadrupole mass spectrometry. Problems due to the occurrence of contamination of the breast milk samples are to be expected for mono(2-ethylhexyl)phthalate (MEHP), the hydrolytic monoester of DEHP, that has shown to also be formed in the matrix due to the residual activity of enzymes (lipases, esterases), even after prolonged storage periods at −20 °C. Therefore, MEHP is a very unreliable parameter for the assessment of DEHP exposure and should not be used for breast milk. In contrast, MECPP, MEHHP, and MEOHP are not susceptible to contamination as they are major metabolites of DEHP created by the liver. They are only formed *in vivo* and serve as a reliable parameter for DEHP exposure. 

The coefficient of variation for the chemicals measured, was 16%–17%. More information on chemical analysis, including limits of quantification and quality control parameters, is given in the Supplemental Material, pp. 2–4. 

### 2.4. Data-Analysis

Data-analysis was performed using IBM SPSS version 20 (Armonk, NY, USA) [35]. For each compound, exposure values below the limit of quantification (LOQ) were replaced by LOQ/√2 [36]. For DDE a conversion factor, based on publications of other European cohorts, was used to transform levels in breast milk to levels in cord blood, as it accumulates in lipid [36]. The following conversion factor was used:

[DDE, cord plasma (ng/L)] = 1.20 × [DDE, breast milk (ng/g lipid)] [36]

For PCB-153, over 50% of the cord plasma samples had an exposure level that could not be quantified. As PCB-153 was determined predominantly in cord blood it was decided to only include cord blood levels of PCB-153 and to dichotomize at LOQ (>LOQ *vs.* <LOQ). For the other compounds no conversion factor was applied and only cord blood levels were used. 

Mixed models were used for the main analysis. For each compound a separate model was composed for weight, height, BMI, and head circumference. Linearity with outcome was checked for each compound, and as none of the compounds showed a linear association, each exposure was split up in quartiles. Exposure quartiles, timing of anthropometric measurement, sex and their interactions were added to the models as fixed effects and a random effect was added for subject. Time was included as a categorical variable. Covariates were selected based on literature and included maternal/paternal BMI and height, birth weight, gestational age, parity, alcohol, smoking, education, and breast feeding duration. As sample size was relatively small, covariates were only added to the models if they were deemed relevant (change in β-coefficient of exposures > 10%). Using this cut-off, birth weight, gestational age, and maternal height influenced the estimates in the majority of analyses and were therefore selected for all models. Sex and time specific marginal means were calculated from the models and were used to plot the development over time in growth parameters for the exposure quartiles and for boys and girls separately. Interactions were considered significant at a *p*-value of 0.10, considering the limited power of this study.

### 2.5. Covariates

Birth weight was measured after birth by a midwife or a nurse and was obtained from registries of the midwives. Newborns were put on the weighing scale without a diaper and birth weight was determined when the infant was in a calm state. Weighing scales were provided by the midwives and were calibrated daily. Gestational age was determined by midwives by means of early ultrasound.

Maternal height was measured by the midwife at inclusion, approximately 10–12 weeks in pregnancy. Midwives received a measuring tape which was attached to a wall in the midwife’s office, as well as instructions on how to perform this measurement, including how the participants should be positioned and the precision required for the measurement. 

## 3. Results

Out of the 89 children included in the analysis, 56 were boys (Table 1). Compared to girls, boys had a higher weight gain between 1 and 11 months of age (5.3 *versus* 4.5 kg), as well as a larger increase in height (21.5 *versus* 19.7 cm) and head circumference (9.0 *versus* 8.1 cm). The majority of the children were breastfed for a period longer than three months (boys: 69.2%, girls: 64.3%). 

An overview of exposure levels of the various compounds is given in Table 2. Information on exposure quartiles is given in Table 3. Due to the high fat content of breast milk samples, wet weight levels of the lipophilic compound DDE were lower than lipid corrected levels. There was no difference in exposures between boys and girls (not shown). 

**Table 1:** Population characteristics of the study cohort.

	Boys (n = 56)	Girls (n = 33)
Weight, age = 1 month (kg)	4.68 (3.00–5.87)	4.25 (3.21–5.38)
Weight, age = 11 months (kg)	10.13 (8.49–12.85)	8.92 (6.88–10.55)
Weight gain, first year (kg)	5.31 (4.16–7.59)	4.46 (3.41–5.60)
Height, age = 1 month (cm)	54.8 (51.0–59.0)	53.9 (51.0–59.9)
Height, age = 11 months (cm)	76.6 (71.0–81.4)	74.6 (71.0–77.0)
Growth, first year (cm)	21.5 (18.0–25.0)	19.7 (16.0–23.3)
BMI, age = 1 month (kg/m^2^)	15.5 (12.8–18.7)	14.8 (12.0–17.0)
BMI, age = 11 months (kg/m^2^)	17.1 (15.6–21.0)	16.2 (13.3–18.5)
BMI change, first year (kg/m2)	1.8 (−1.6–5.2)	1.6 (−0.8–3.2)
Head circumference, age = 1 month (cm)	38.0 (34.2–40.6)	37.3 (35.1–40.1)
Head circumference, age = 11 months (cm)	46.8 (43.4–49.5)	45.2 (42.6–48.0)
Head circumference change, first year (cm)	9.0 (7.9–11.2)	8.1 (6.7–10.4)
Birth weight (g)	3624.5 (491.7)	3564.2 (397.5)
Gestational age (weeks)	39.7 (1.5)	40.1 (1.0)
Parity (nulliparous, %)	23 (42.6%)	10 (30.3%)
Height mother (cm)	171.4 (5.7)	171.9 (4.6)
Height father (cm)	185.2 (6.8)	183.6 (6.2)
BMI mother (start pregnancy, kg/m^2^)	23.6 (3.3)	23.0 (4.2)
BMI father (kg/m^2^)	24.0 (3.0)	25.0 (2.8)
Age mother (years)	30.3 (3.8)	31.9 (3.5)
Age father (years)	32.4 (5.6)	34.2 (4.2)
Breastfeeding		
No breastfeeding	4 (7.7%)	3 (10.7%)
0–3 months breastfeeding	12 (23.1%)	7 (25.0%)
>3 months breastfeeding	36 (69.2%)	18 (64.3%)
Education mother (bachelor/master, %)	41 (75.9%)	19 (59.4%)
Smoking (first trimester, yes, %)	2 (3.7%)	2 (6.1%)
Alcohol (first trimester, yes, %)	3 (2.7%)	2 (6.6%)

Unless stated otherwise, values are median (range) or mean ± SD.

**Table 2:** Exposure profile of the study cohort.

Compound		n	Mean	Median	Range	LOQ	<LOQ (%)
PCB-153							
Cord plasma	ng/L	52	35.94	29.14	22.63–96.00	21–43	55.8
	ng/g lipid	52	36.31	31.04	17.95–88.89	14–53	55.8
DDE							
Cord plasma	ng/L	52	115.61	83.00	28.28–470.00	33–73	23.1
	ng/g lipid	52	116.16	82.06	28.83–580.25	23–86	23.1
Breast milk	ng/L	24	2527.92	1950.00	400.00–1,1390.00	9.20–13.00	0
	ng/g lipid	24	62.58	45.33	12.11–277.80	0.13–0.53	0
Total ^a^	ng/L	76	102.82	74.50	14.53–470.00		15.8
MECPP							
Cord plasma	ng/mL	61	0.31	0.27	0.11–1.00	0.13–0.28	6.6
MEHHP							
Cord plasma	ng/mL	61	0.32	0.26	0.10–1.00	0.14–0.27	11.5
MEOHP							
Cord plasma	ng/mL	61	0.29	0.22	0.12–0.87	0.17–0.33	26.2
PFOA							
Cord plasma	ng/L	61	940.2	870.0	300–2700	50–140	0
PFOS							
Cord plasma	ng/L	61	1611.0	1600.0	570–3200	44–140	0

^a^ For total DDE, cord plasma exposure data were merged with breast milk exposure levels converted to cord plasma levels.

**Table 3:** Exposure quartiles.

Compound	Q1	n	Q2	n	Q3	n	Q4	n
DDE (Total) (ng/L)	<44.74	19	44.74–74.50	19	74.51–113.11	19	>113.11	19
MECPP (ng/mL)	<0.16	15	016–0.24	15	0.25–0.34	15	>0.34	15
MEHHP (ng/mL)	<0.20	16	0.20–0.23	17	0.24–0.37	11	>0.37	17
MEOHP (ng/mL)	<0.13	17	0.13–0.20	17	0.21–0.34	13	>0.34	14
PFOA (ng/L)	<591	17	591–870	14	871–1100	15	>1100	15
PFOS (ng/L)	<1001	18	1001–1600	15	1601–2000	14	>2000	14

PCB-153 was included as a dichotomous variable (<LOQ *vs.* >LOQ).

### 3.1. BMI

**Figure 1:**
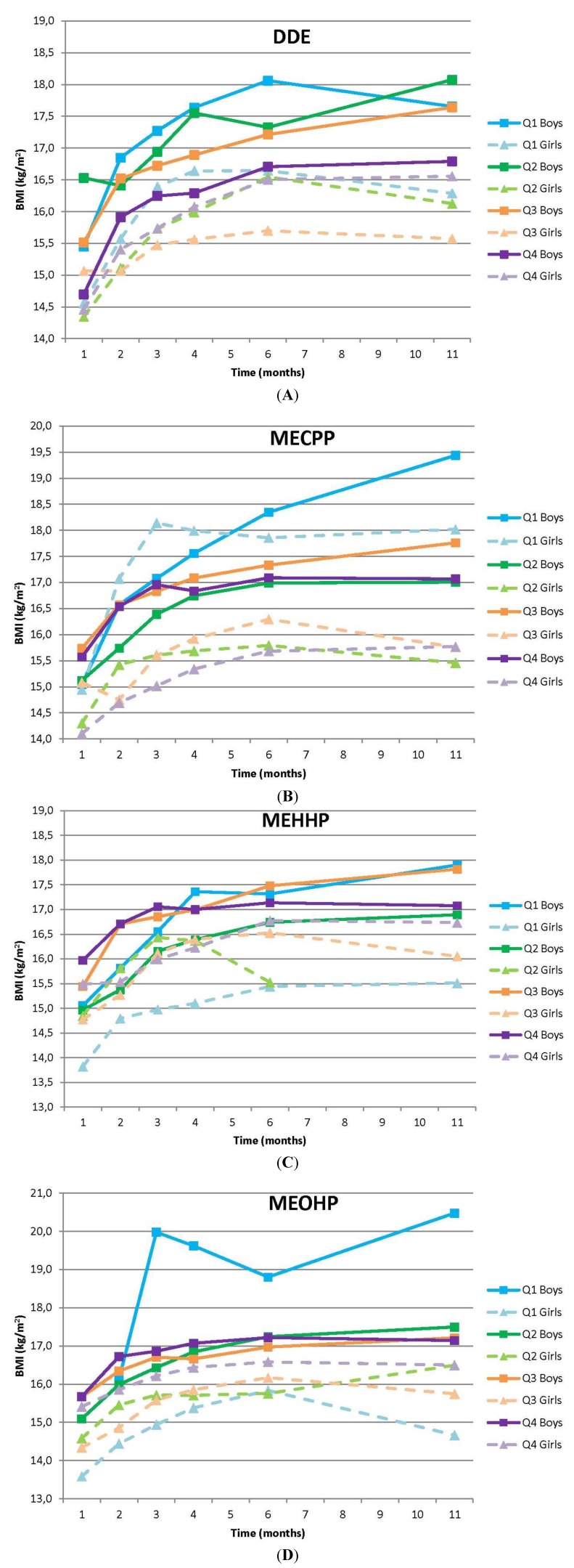
Sex specific BMI curves for early life DDE (**A**), MECPP (**B**), MEHHP (**C**) and MEOHP (**D**) exposure.

Sex-specific BMI curves for DDE, MECPP, MEHHP, and MEOHP are given in Figure 1A–D. For the other EDCs they can be found in the Supplemental Material pp. 5–6. For all compounds, significant main effects were observed for both time and sex, but not for compound (Table 4). We did find a relevant main effect for MECCP which was 1.5 and 2.2 at 11 months for boys and girls respectively (*p* = 0.051). Interaction effects were observed for total DDE and MEOHP. 

Total DDE showed an interaction with time, independent of sex, meaning that BMI differed between exposure quartiles over time (*p* = 0.078). Boys in the highest DDE exposure quartile had a BMI which was lower than boys with a lower DDE exposure at all ages (Figure 1A). At the age of 11 months, the difference with the nearest quartile reached 0.8 kg/m^2^ (mean BMI Q4: 16.8 kg/m^2^, 95% CI 16.09 to 17.49; mean BMI Q3: 17.6 kg/m^2^, 95% CI 16.93 to 18.35). A similar result was observed for girls in the third exposure quartile.

For MEOHP a significant interaction with time and sex on BMI was observed (*p* = 0.029), indicating that BMI differed between exposure quartiles during the first year, and that these patterns were different for boys and girls (Figure 1D). In boys, weight at one month of age was similar for all quartiles. However, from 3 months onwards, boys in the lowest exposure quartile showed a very deviant development in their BMI from all other boys and girls in the sample. On average their BMI was much higher. 

**Table 4:** *p*-values of tests of main effects and interactions of compound with time, sex, or time and sex, for each health outcome.

EDC	Effect	BMI	Weight	Height	Head Circumference
		*p*	*p*	*p*	*p*
DDE	Time	<0.001 *	<0.001 *	<0.001 *	<0.001 *
	DDE	0.293	0.647	0.910	0.778
	Sex	<0.001 *	<0.001 *	0.024 *	<0.001 *
	Time × DDE	0.078 *	0.84	0.490	0.721
	DDE × sex	0.420	0.205	0.484	0.905
	Time × DDE × sex	0.298	0.695	0.134	<0.001 *
PFOS	Time	<0.001 *	<0.001 *	<0.001 *	<0.001 *
	PFOS	0.586	0.802	0.975	0.649
	Sex	<0.001 *	<0.001 *	0.040 *	<0.001 *
	Time × PFOS	0.878	0.910	0.485	0.800
	PFOS × sex	0.655	0.425	0.749	0.485
	Time × PFOS × sex	0.679	0.617	0.321	0.742
PFOA	Time	<0.001 *	<0.001 *	<0.001 *	<0.001 *
	PFOA	0.813	0.350	0.045 *	0.774
	Sex	<0.001 *	<0.001 *	0.019 *	<0.001 *
	Time × PFOA	0.389	0.126	<0.001 *	0.001 *
	PFOA × sex	0.242	0.169	0.165	0.467
	Time × PFOA × sex	0.348	0.812	0.152	0.387
MECPP	Time	<0.001 *	<0.001 *	<0.001 *	<0.001 *
	MECPP	0.051	0.265	0.726	0.314
	Sex	0.004 *	<0.001 *	0.002 *	0.003 *
	Time × MECPP	0.117	0.090 *	0.944	0.604
	MECPP × sex	0.496	0.277	0.185	0.047 *
	Time × MECPP × sex	0.204	0.929	0.978	0.886
MEHHP	Time	<0.001 *	<0.001	<0.001 *	<0.001 *
	MEHHP	0.279	0.204	0.133	0.144
	Sex	0.005 *	0.002	0.163	0.012 *
	Time × MEHHP	0.593	0.962	0.294	0.100 *
	MEHHP × sex	0.383	0.326	0.073 *	0.197
	Time × MEHHP × sex	0.127	0.485	0.011 *	0.018 *
MEOHP	Time	<0.001 *	<0.001 *	<0.001 *	<0.001 *
	MEOHP	0.315	0.151	0.112	0.119
	Sex	<0.001 *	0.001 *	0.390	<0.001 *
	Time × MEOHP	0.152	0.948	0.084 *	0.335
	MEOHP × sex	0.104	0.831	0.207	0.253
	Time × MEOHP × sex	0.029 *	0.037 *	<0.001 *	0.240
PCB-153 (>LOQ *vs.* <LOQ)	Time	<0.001 *	<0.001 *	<0.001 *	<0.001 *
	PCB	0.203	0.544	0.598	0.729
	Sex	<0.001 *	<0.001 *	0.001 *	<0.001 *
	Time × PCB	0.491	0.518	0.946	0.861
	PCB × sex	0.338	0.738	0.115	0.586
	Time × PCB × sex	0.808	0.775	0.086 *	0.102

* Main effects considered significant at *p* ≤ 0.05, interaction effects at *p* ≤ 0.10.

### 3.2. Weight and Height

Sex specific weight and height curves for each compound can be found in the Supplemental Material pp. 7–14. Main effects of time and sex were significant for all compounds for both weight and height (Table 4). No main effects of exposure were observed, except for PFOA and height. For MEOHP results regarding weight and height were similar to BMI, showing a significant interaction with both time and sex in a similar direction. 

### 3.3. Head Circumference

**Figure 2:**
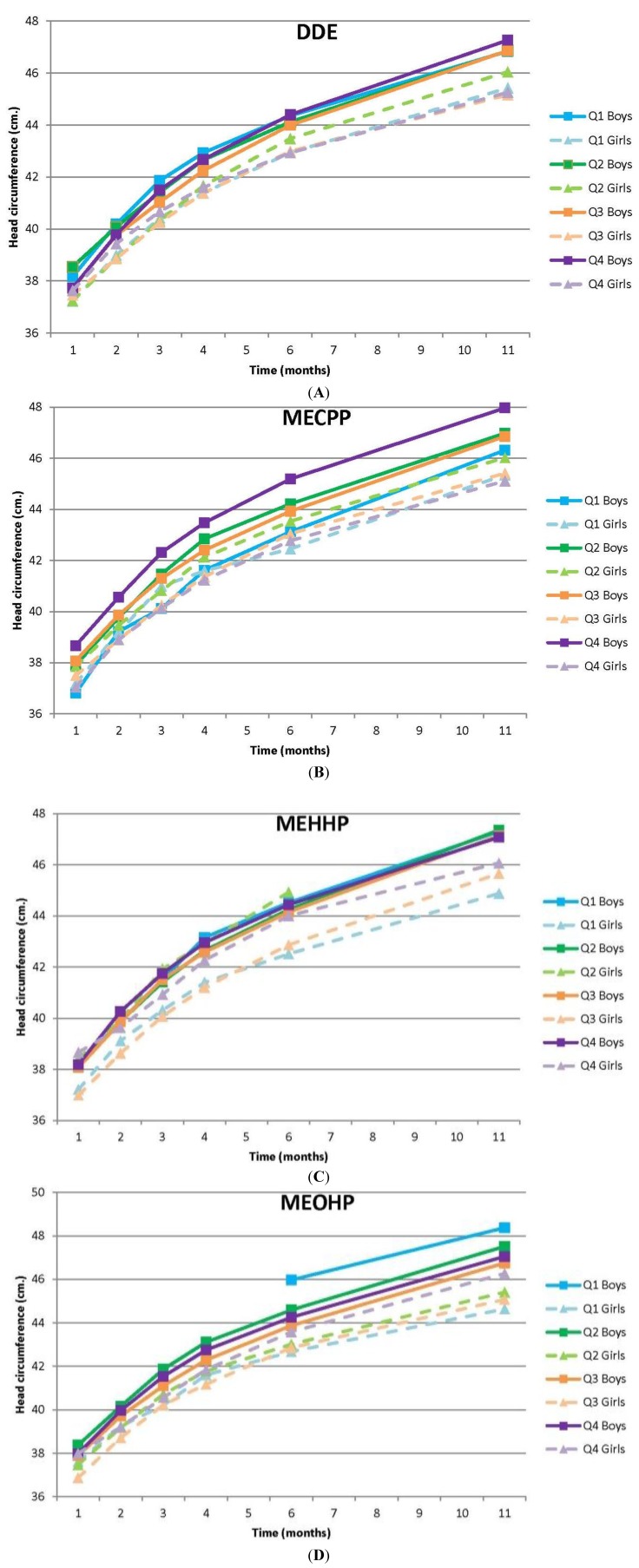
Sex specific head circumference curves for early life DDE (**A**), MECPP (**B**), MEHHP (**C**) and MEOHP (**D**) exposure.

Sex-specific head circumference curves for DDE, MECPP, MEHHP, and MEOHP are given in Figure 2A–D. For the other EDCs they can be found in the Supplemental Material pp. 15–16. Similar to BMI, main effects for both time and sex on head circumference were significant for all compounds, whereas no main effects for exposure were observed (Table 4). Interaction effects were observed for total DDE, PFOA, MECPP, and MEHHP. 

For total DDE a significant interaction with time and sex on head circumference was observed (*p* < 0.001), indicating that head circumference differed between exposure quartiles during the first year, and that these patterns were different for boys and girls. Girls in the second quartile of exposure had a greater head circumference from six months onwards. Boys in the highest exposure quartile showed a similar pattern.

Boys in the highest MECPP exposure quartile had a consistently greater circumference over time than the other boys (Figure 2B). Those in the lowest quartile on the other hand had a consistently smaller head circumference than the other quartiles. There was also a significant interaction for MEHHP exposure with time and sex (*p* = 0.018). In girls, the second exposure quartile in particular showed a consistently greater head circumference over time compared to the other quartiles (Figure 2C). At six months of age the difference compared to the two quartiles showing the smallest head circumference was 2.0 cm, which was not statistically significant (head circumference Q2: 44.9 cm, 95% CI 43.2 to 46.7; head circumference Q3: 42.9 cm, 95% CI 42.2 to 43.5).

## 4. Discussion

The objective of this study was to determine the possible associations between early life exposure to various EDCs and child growth in the first year of life. This is, to our knowledge, the first study to examine prenatal phthalate exposure in relation to growth in early childhood. Importantly, in our study we have measured phthalate metabolites in cord blood, whereas most studies use maternal urine as a proxy for prenatal exposure. Although our sample was relatively small, our analyses revealed some potentially relevant findings. Phthalate metabolites were associated with both BMI and head circumference, with low exposure levels associated with higher BMI values, and high exposure levels associated with a greater head circumference. DDE exposure was also associated with BMI and head circumference, however patterns were less consistent. None of the compounds showed a linear dose-response relation with the outcomes included.

For all growth parameters effects of DEHP exposure were observed. Low exposure levels in particular were associated with higher BMI values. We furthermore observed continuous increases in BMI between 6 and 11 months of age for boys in the first and third exposure quartiles of all DEHP metabolites. Similar early life growth patterns have been observed for children who were overweight or obese when five years old, as observed in the study by Gittner *et al.* [9]. Reference BMI curves of the World Health Organization generally show a decline in BMI in the second half of the first year [37]. In this cohort, girls did show a stabilization of or a decline in BMI during the last six months. These results suggest that prenatal DEHP exposure may possibly predispose children, and perhaps boys in particular, to a more obese growth pattern. However, these results do need to be replicated in larger studies with more longitudinal follow-up as numbers in the current study are very low. Experimental studies have also indicated that maternal DEHP exposure in rodents leads to increases in body weight in offspring [38]. The biological mechanism underlying DEHP effects on obesity has been proposed to be through its activation of peroxisome proliferator-activated receptors (PPAR), master regulatory genes in lipid metabolism and adipocyte differentiation [38,39].

Early life exposure to phthalates was also related to changes in head circumference during the first year. In particular in boys and girls with the highest exposure to respectively MECPP and MEHHP exposure a higher head circumference was observed during the first year. We could not find any other studies which investigated this, except for a few papers which related prenatal exposure to head circumference at birth, but none of those showed any significant effect on head circumference at birth for MECPP, MEHHP, or MEOHP [40,41,42,43]. For MEHHP exposure, we observed a range of 2 cm across exposure quartiles at 6 months of age, and even though this difference was not significant, the absolute difference in itself is quite large as the range between P10 and P90 at this age in girls is 3.4 cm according to the WHO reference tables [44]. Follow-up of these children is essential to see if these variations persist throughout childhood and to clarify whether these results are relevant regarding neurodevelopment.

For DDE, a difference in BMI between exposure quartiles over time was observed. Boys highest exposed to DDE had a consistently lower BMI compared to their peers in the other quartiles, and for girls the third exposure quartile in particular showed less increase in BMI over time compared to the other girls. Several studies have reported no effect of prenatal DDE exposure on growth, including a cohort of Mexican boys with a median age of 18 months at follow-up [45], as well as another Mexican cohort with follow-up until 12 months of age [15]. DDE exposure in both these cohorts was however much higher than in our sample. Interestingly, however, our study indicated that lower exposures to DDE were associated with an increase in BMI between 6 and 11 months of age. Similarly, in the Spanish INMA cohort, with DDE levels similar to this cohort, prenatal exposure was associated with rapid weight gain in the first six months, and a higher BMI at the age of 14 months [10]. In Belgian children, prenatal DDE exposure was associated positively with BMI in 3-year-olds [27]. Animal studies have indicated that long term, multigenerational exposure to DDT results in weight gain in offspring, though biological mechanisms have not been elucidated [46].

Effects for PCB-153, PFOS, and PFOA were less evident. For PCB-153 changes in height were significant over time and sex. These differences between groups were however only visible during the first three months, after which results became similar for all groups. For the other growth parameters no effect was observed. For head circumference a deviation for PCB-153 curves was observed compared to curves for other compounds, which showed a continuous increase in head circumference over time, while a decrease was observed for PCB-153 between four and six months of age. This is probably due to variations in the number of participants, which differs across quartiles as well as between compounds. The decrease was however insignificant.

For PFOS, no significant interaction effects were shown, and even though for PFOA exposure quartiles developed differently over time regarding height and head circumference, variation between quartiles was relatively small. Studies regarding prenatal PFAA exposure and childhood anthropometry are scarce. Halldorsson *et al.* showed a positive association with BMI for 20-year-old women who were prenatally exposed to PFOA [14]. In the Danish National Birth Cohort inverse associations with BMI were seen for both PFOS and PFOA when the children were twelve months of age [47], however at seven years of age these associations were no longer apparent [18]. More research is required to elucidate whether prenatal PFAA exposure affects growth in children.

Results of the current study showed some sex specific effects, which have also been observed in some previous studies. In a cohort of 7-year-old children from the Faroe Islands, associations between prenatal PCB and DDE exposure and BMI were only observed in girls, and in particular in girls who had mothers with a high pre-pregnancy BMI [48]. In the INMA cohort, higher exposures to DDE were related to an increased risk for overweight, and this result was strongest in girls [12]. Higher DDT exposure on the other hand, was associated with an increased risk for being overweight in boys only. As mentioned before, studies on prenatal phthalate exposure and obesity/overweight are not available, however in a cross-sectional study, Hatch *et al.* reported that exposure to various phthalates, as determined in urine, was positively associated to BMI in males aged 20–59 years, and in females aged 12–19 years [49]. Not all studies report results stratified for sex, and as these compounds are suspected to disrupt the endocrine system—which is specific for males and females—future studies should aim to include sex specificity in their results. Further experimental research would also be important to understand mechanisms underlying the sex specific effects of EDCs. 

Various mechanisms are possible through which early life exposure to EDCs may affect anthropometry in a sex-specific fashion. Most EDCs are thought to have (anti-)estrogenic or (anti)androgenic properties. Studies in sheep have shown that prenatal exposure to testosterone is associated with lower birth weight, developmental changes in insulin-like growth factor (IGF)/IGF binding protein system, and insulin resistance (reviewed in [50]). In a recent study in which rats were developmentally exposed to the suspected androgen DEHP, female rats were observed to have impaired glucose tolerance and insulin secretion [51]. Male rats showed increased serum insulin levels, and both female and male rats had a significantly lower birth weight than controls. Environmental exposure to chemicals may also affect anthropometry through interference with glucocorticoids. Increased maternal glucocorticoid levels have been associated with lower birth weight and altered set-point of the hypothalamic-pituitary-adrenal (HPA) axis (reviewed in [52]). Hydroxy-PCBs have been shown to have anti-glucocorticoid properties in human placenta [53], whereas in adipocytes BPA and dicyclohexyl phthalate (DCHP) activated the glucocorticoid receptor and increased lipid accumulation [54]. 

None of the compounds included showed a linear dose-response relation with any of the growth outcomes. This has however also been reported by other studies on early life EDC exposure and growth [14,16,55]. Nonmonotonic dose-response relations have been reviewed by VandenBerg *et al.* (2012), who indicate that the endocrine system is designed to work at low concentrations and that low dose effects cannot be predicted from effects observed at higher doses [25]. Whether the associations we observe are truly non-monotonic remains to be clarified by larger studies.

Models were adjusted for birth weight, as birth weight in itself may predispose individuals to a more obese growth pattern. However, several studies have shown that prenatal exposure to certain EDCs is associated with birth weight (see the meta-analysis by Govarts *et al.* [36]) and therefore birth weight may also be in the causal pathway between exposure and childhood obesity. Including this factor in the model would then lead to overcorrection. We therefore performed a sensitivity analysis for BMI without birth weight in the models (not shown), which did not affect any of the interaction effects, except between DDE and sex, which became significant, but no changes were observed in terms of relevance. For boys in the first quartile of PFOA exposure, and the third quartile of both PFOS and MEHHP exposure, a higher BMI was observed compared to the other groups if birth weight was not included in the models. These quartiles where however also relatively high when birth weight was included in the models.

A limitation of this study is the small sample size. As associations between outcomes and exposures were not linear, participants were divided in quartiles based on exposure levels. This also reduced the power. In addition, cut points were therefore not based on clinical relevance which might have obscured real effects or evoked non-relevant effects. However, no accepted cut-off level exists to our knowledge. Moreover we stratified results for sex, which reduced power of the tests. However, as growth differs between boys and girls and the chemicals in question may affect the endocrine system, stratification was necessary. Finally, our inclusion of covariates in the model could have potentially further reduced power. However standard errors were not affected by including them into the models. They were therefore included in order to reduce bias by confounding as much as possible. Another limitation results from the fact that we performed multiple tests which has inflated the risk of type I errors. We have chosen not to adjust our alpha for multiple testing, as this would further reduce the power of our statistical tests, but acknowledge that some of our significant findings may very well be false positive findings. 

The strengths of the study are the prospective data collection, the large range of compounds included, and the fact that the cohort was very homogenous. Associations are therefore less likely to be confounded by demographic or socio-economic factors. The BMI of children, when four weeks old, was slightly higher compared to the general Dutch, Caucasian population. According to the fifth National Growth Study performed in 2009, median BMI at this age was 14.75 for boys, and 14.55 for girls, compared to 15.53, and 14.83 for boys and girls respectively in the current study [56]. In one-year-olds, median BMI was 17.18 in boys, and 16.76 in girls in the general population, compared to respectively 17.14, and 16.21 in our cohort, indicating that girls had a slightly lower BMI at this age than would be expected. Furthermore, mothers in this cohort have more often received higher education compared to the general Dutch population [57], and the sex-ratio (1.7) of the offspring is higher than in general. Furthermore the male/female ratio of offspring was 1.7, which is higher than average. We have no explanation for this, as the cohort was recruited prospectively. There is no indication that early life exposure to EDCs alter male/female ratio [58]. Considering these factors, extrapolation of results to other populations should be performed with caution.

## 5. Conclusions

In conclusion, based on our study on first year growth, low exposure to phthalates may be associated with a higher BMI over time, which was reflected in both higher weight and height. Furthermore there may be an inverse association between DDE exposure and BMI. Results from our study should be interpreted with caution due to the limited sample size. Therefore confirmation of our results in larger studies is warranted. In addition there is a need for a longer follow-up to examine whether these associations persist into later childhood. 

## Supplementary Files

Supplementary File 1 Supplementary Material (PDF, 4642 KB)
