# Supplementary material for: First Year Growth in Relation to Prenatal Exposure to Endocrine Disruptors — A Dutch Prospective Cohort Study
Source: Int J Environ Res Public Health. 2014 Jul 10;11(7):7001–21. doi: 10.3390/ijerph110707001 (PMC4113857; doi:10.3390/ijerph110707001)
Supplement: Supplementary File 1 — Supplementary Material (PDF, 4642 KB) [file ijerph-11-07001-s001.pdf]

Supplementary Material

First Year Growth in Relation to Prenatal Exposure to Endocrine Disruptors — A Dutch Prospective Cohort Study

Contents

Analysis of Organochlorine Pesticides and PCB 153 2
Analysis of DEHP Metabolites 2
Analysis of PFOS and PFOA 3
Determination of Lipid Content of Breast Milk and Cord Plasma Samples 3
QA/QC Procedures 4
Figure S1. Sex specific BMI curves for early life PFOS (A), PFOA (B) and PCB-153 (C) exposure. 5
Figure S2. Sex specific weight curves for early life DDE (A), PCB-153 (B), PFOS (C), PFOA (D), MEHHP (E), MEOHP (F) and MECPP (G) exposure. 7
Figure S3. Sex specific height curves for early life DDE (A), PCB-153 (B), PFOS (C), PFOA (D), MEHHP (E), MEOHP (F) and MECPP (G) exposure. 11
Figure S4. Sex specific head circumference curves for early life PFOS (A), PFOA (B) and PCB-153 (C) exposure. 15
References 16

Analysis of Organochlorine Pesticides and PCB 153

For the determination of the organochlorine pesticide p,p'-DDE and PCBs (PCB153), 3 mL of cord plasma or 12 mL breast milk were dried with Kieselguhr after addition of the internal standard ($^{13}C_{10}$-PCB153 (from Cambridge Isotope Laboratories) BDE58). The dried samples were extracted with 30 mL hexane/dichloromethane (7:3 v/v) for 30 min by sonication. The extracts were purified over sulphuric acid silica columns (5 g, 40% sulphuric acid w/w) that were eluted with 30 mL hexane/dichloromethane (7:3 v/v). The eluates were evaporated under a gentle stream of nitrogen. Finally, the extracts were quantitatively transferred to GC-vials, with a final volume of 100 µL iso-octane.

The samples were analyzed using an Agilent 6890 GC with a 5975 Mass Spectrometric Detector in negative chemical ionization mode. The GC was equipped with a CPSil-8 CB column (Varian, 50 m × 0.25 mm ID × 0.25 µm film thickness). The samples were injected in pulsed splitless mode with the injector at 275 °C. The oven was programmed as follows: initial temperature: 90 °C for 3 min; then to 210 °C at 30 °C/min and held for 20 min; finally to 290 °C at 5 °C/min and held for 3 min (total run time 45 min). Carrier gas was helium at a flow rate of 2.7 mL/min. The compounds of interest were quantified by using *m/z* 359.8 for PCB 153 and *m/z* 35 for p,p'-DDE (compound confirmation by GC-MS/EI measurement of *m/z* 246).

Analysis of DEHP Metabolites

To assess the exposure to DEHP, the secondary metabolites MEOHP (mono(2-ethyl-5-oxohexyl) phthalate), MEHHP (mono (2-ethyl-5-hydroxyhexyl) phthalate) and MECPP (mono(2-ethyl-5-carboxypentyl) phthalate) were quantitatively determined. The remaining enzymatic activity in the sample material was quenched by adding 0.02 mL 1 M phosphorous acid to 0.3 mL cord plasma or 0.04 mL 1 M phosphorous acid to 0.5 mL breast milk. The mixtures were then sonicated for 5 min. To adjust the pH of the plasma and milk samples to 6.2, 0.04 mL and 0.06 mL 1M NaOH was added to the samples, respectively. After the addition of the internal standard ($^{13}C_4$-MEOHP, $^{13}C_4$-MEHHP, $^{13}C_4$-MECPP and MEHP- d_4, all from Cambridge Isotope Laboratories), 5 µL β–glucuronidase from *E. Coli* K12 (from Roche) in 0.2 mL 2.5 M ammonium acetate buffer (pH 6.2) was added. The samples were incubated for 90 min at 37 °C. The completeness of the deconjugation step was checked by adding 4-methylumbelliferone-glucuronide to each sample. The β–glucuronidase activity was stopped by adding 0.06 mL formic acid to the plasma samples and 0.06 mL ammonium hydroxide to the milk samples followed by sonication for 15 min and overnight storage at −20 °C.

The deconjugated plasma samples were thawed and centrifuged for 15 min at 17,000 rpm. The supernatants are transferred to a vial to which 0.2 mL water is added.

The milk samples were also centrifuged for 15 min (17,000 rpm) to remove the lipids. The remaining part was extracted by solid phase extraction (SPE). The Oasis MAX 3 cc 60 mg cartridges were conditioned with 3 mL methanol and 3 mL milliQ. After the samples were loaded at 1 mL/min, the cartridges were washed with 1 mL 5% ammonium hydroxide and 1 mL 75% tetrahydrofuran in methanol. The metabolites were eluted from the cartridges with 5 mL 5% formic acid in methanol. To the eluates 0.2 mL milliQ was added before evaporation to a volume of 0.2 mL. Finally, 0.2 mL 4% ammonium hydroxide and 0.2 mL milliQ were added to the extracts. The obtained extracts were injected onto a RAM (restricted access material) phase cartridge (LiChrospher RP-8 ADS, 25 µm,

25 × 4 mm). After trapping and cleanup, the analytes were eluted in backflush mode and transferred to the analytical column (Luna Phenyl-hexyl 75 × 4.6 mm) using a gradient of 0.1% acetic acid and acetonitrile with a flow rate of 0.25 mL/min. The LC system was an Agilent 1200 Series (Palo Alto, CA, USA) coupled with an Agilent 6410 electro spray interface (ESI) operated in the negative ion mode prior to triple-quadrupole mass spectrometric detection. For MECPP, the ion transition used for quantification was *m/z* 307.1–*m/z* 159.1, for MEHHP the ion transition *m/z* 293.1–*m/z* 145.1, for MEOHP the ion transition *m/z* 291.1–*m/z* 143.1, and for MEHP the ion transition *m/z* 277.1–*m/z* 134.1 were used.

Analysis of PFOS and PFOA

For the determination of perfluorooctane sulfonate (PFOS) and perfluorooctanoic acid (PFOA), the breast milk samples were thawed and homogenized after stabilizing them at a temperature of 38 °C. Subsequently, aliquots of 0.5 mL of each sample were taken for analysis. The sample was sonicated for 30 min after addition of the internal standards ($^{13}C_4$-PFOA and $^{13}C_4$PFOS, from Wellington Laboratories) and 0.5 mL 1M formic acid. Solid phase extraction was carried out using 1 cc, 30 mg Oasis Wax cartridges. The cartridges were conditioned with 1 mL methanol and 1 mL MilliQ. The samples were loaded at a flow rate of 1 mL/min along with the rinse volume of the sample tube, *i.e.*, 1 mL 25 mM ammonium acetate pH4. The cartridges were washed with 1 mL 25 mM ammonium acetate pH4 and 0.5 mL 25% tetrahydrofuran in methanol. The PFASs were eluted from the cartridge with 0.4 mL 1% NH_4OH in methanol and 0.4 mL 0.1 M formic acid was added to the eluate.

For the analysis of the cord plasma samples 0.2 mL cord plasma was mixed with 0.2 mL methanol. After addition of the internal standard the mixture was homogenized and centrifuged for 15 min at 17,000 rpm. The obtained supernatants were diluted and mixed with 0.5 mL 0.1 M formic acid.

The total volume of the extracts was injected and the PFASs are trapped on a C8-column (Xterra MS C_8, 10 mm × 4.6 mm, particle size 5 µm) in an on-line system with the analytical column (Betasil C8, 50 mm × 2.1 mm, particle size 3 µm). Subsequently the PFASs are eluted from the trapping column and separated on the analytical column using gradient elution at a flow rate of 0.3 mL/min. For the gradient 20 mM NH_4AC pH4 and acetonitrile were used. The LC system was an Agilent 1200 Series (Palo Alto, CA, USA) coupled with an Agilent 6410 electro spray interface (ESI) operated in the negative ion mode prior to triple-quadrupole mass spectrometric detection. For PFOA, the ion transition used for quantification was *m/z* 413–*m/z* 369, and for PFOS the ion transition *m/z* 499–*m/z* 80 was used.

Determination of Lipid Content of Breast Milk and Cord Plasma Samples

In the breast milk samples, the lipid content was determined using a method adapted from Manirakiza *et al.* (1). To 6 mL of sample, 13 mL of isoproanol and 15 mL of cyclohexane were added. The mixture was shaken vigorously for 5 min. Subsequently, 10 mL water was added. The cyclohexane phase containing the lipids was separated from the mixture. This procedure was repeated with a mixture of 15 mL isopropanol and cyclohexane (13:87 v/v). After combining the two cyclohexane fractions, the solvent was evaporated by a gentle nitrogen stream till dryness. The remaining lipids were gravimetrically determined after drying for 1 h at 105 °C.

In cord plasma, the lipid content was determined using standard protocols by the measurement of the triglycerides and cholesterol at the clinical laboratory of the academic hospital (ISO 15189 accredited) of the VU University, VUmc (Amsterdam, The Netherlands).

QA/QC Procedures

For all the analyses described, no Certified Reference Materials (CRMs) were available. Therefore, in every measurement series (<16 samples) a procedure blank, an enriched sample (similar/same matrix) and a sample from a previous series were included. The analytical values obtained for the enriched sample should fall within 20% of the known level. The re-analysis of a sample from a previous series, should give a result with *z*-values < |2|.

In case the procedure blank revealed that the compound(s) to be analyzed were present above the limit of detection (LOD), the series was repeated. All results were corrected using the long term average blank value. The obtained blank data were used for the determination of the LOD (as 3* standard deviation in the blank) and the limit of quantitation (LOQ, defined as 3*LOD).

The performance characteristics of all methods—LOD, recovery and repeatability for breast milk and cord plasma—are given in Table S1.

Table S1. Performance characteristics of all the methods used for the assessment of exposure markers in breast milk and cord plasma.

	**Milk**			**Plasma**		
	**LOD (pg/mL)**	**Recovery (%)**	**Repeatability (%)**	**LOD (pg/mL)**	**Recovery (%)**	**Repeatability (%)**
PCB153	10	96 (89–105)	7	13	105 (97–113)	5
4,4'-DDE	17	98 (91–109)	9	13	96 (82–119)	13
PFOS	1.4	105 (89–105)	9	5	86 (81–90)	3
PFOA	0.4	102 (98–104)	8	7	84 (75–90)	6
MECPP	20	94 (84–104)	5	40	100 (96–106)	3
MEHHP	30	95 (78–107)	11	10	92 (81–89)	4
MEOHP	30	91 (67–113)	16	20	92 (82–98)	6
MEHP	60	96 (65–120)	16	30	75 (70–83)	5

Figure S1. Sex specific BMI curves for early life PFOS (**A**), PFOA (**B**) and PCB-153 (**C**) exposure.

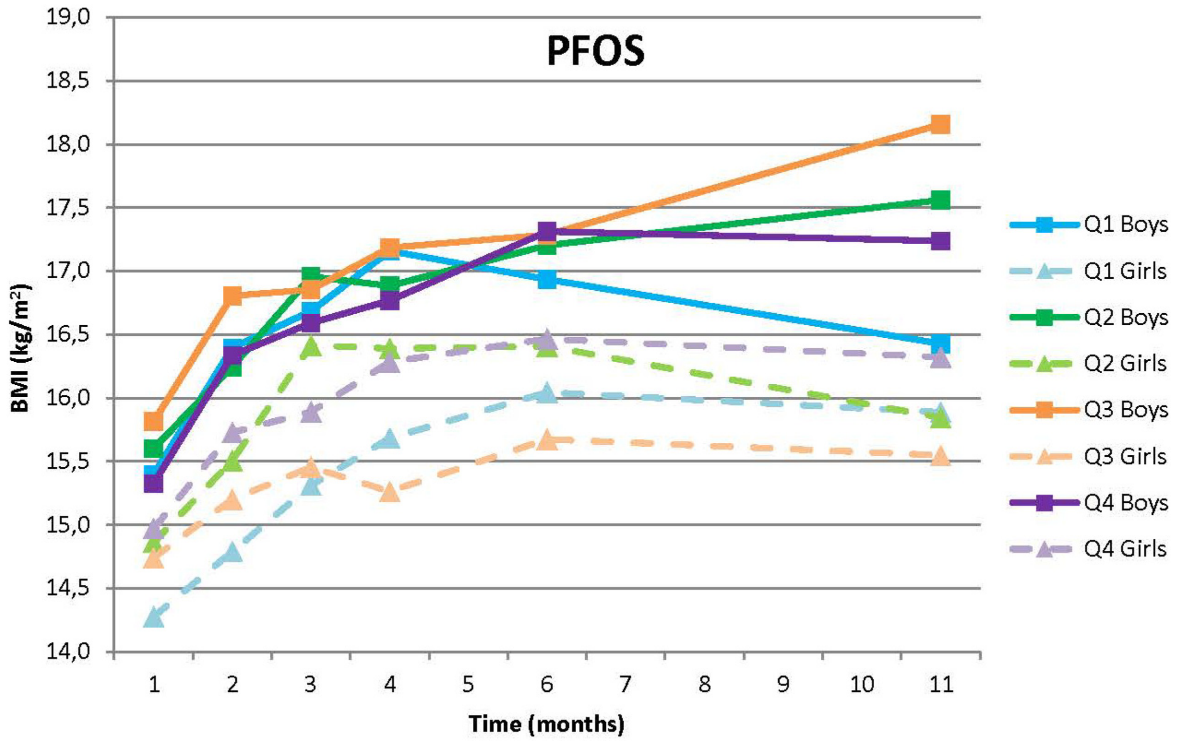

(**A**)

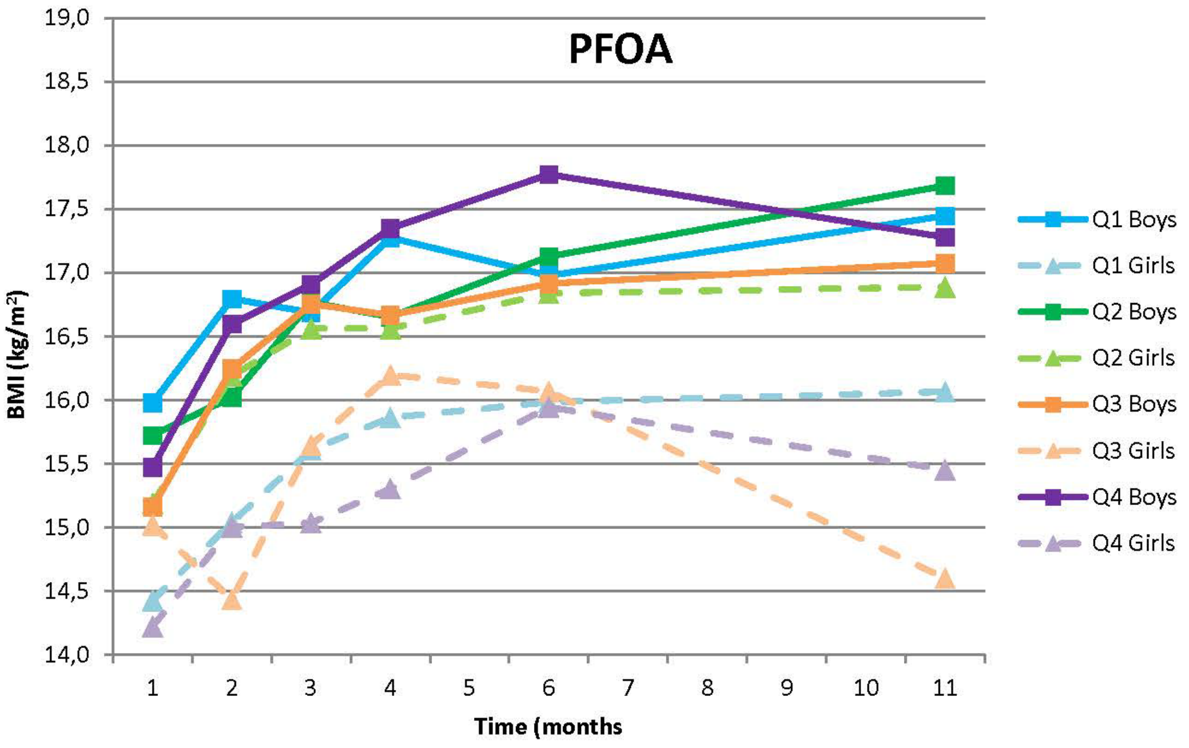

(**B**)

Figure S1. *Cont.*

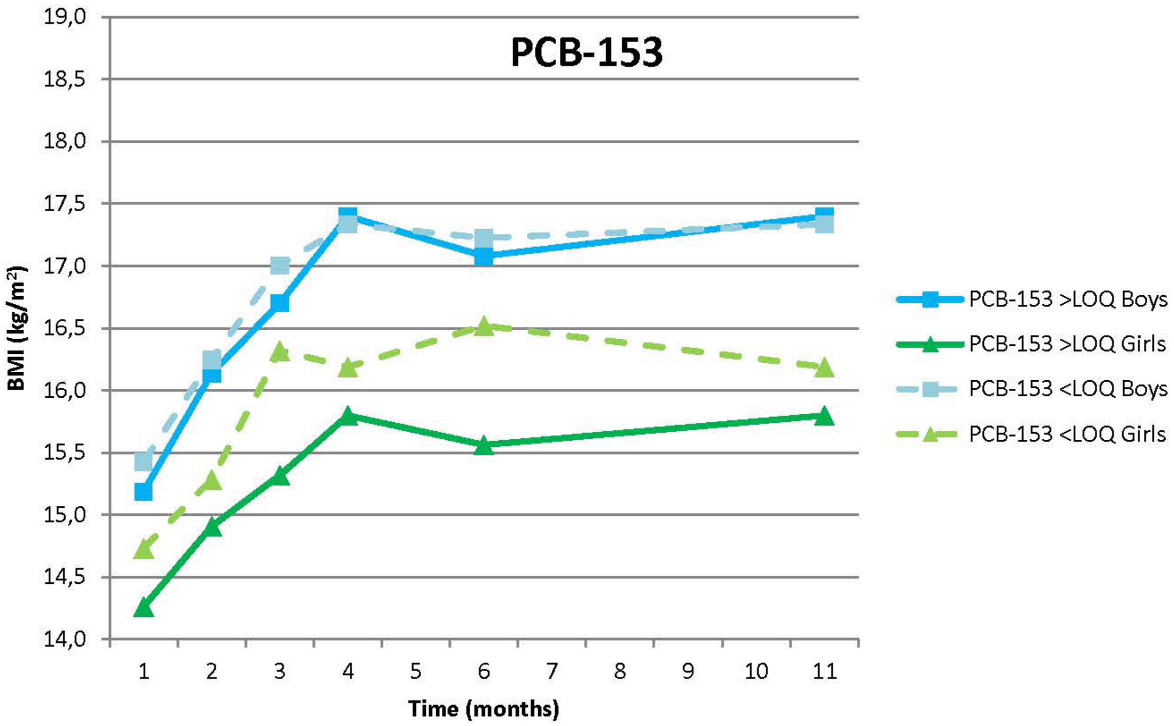

(**C**)

Figure S2. Sex specific weight curves for early life DDE (**A**), PCB-153 (**B**), PFOS (**C**), PFOA (**D**), MEHHP (**E**), MEOHP (**F**) and MECPP (**G**) exposure.

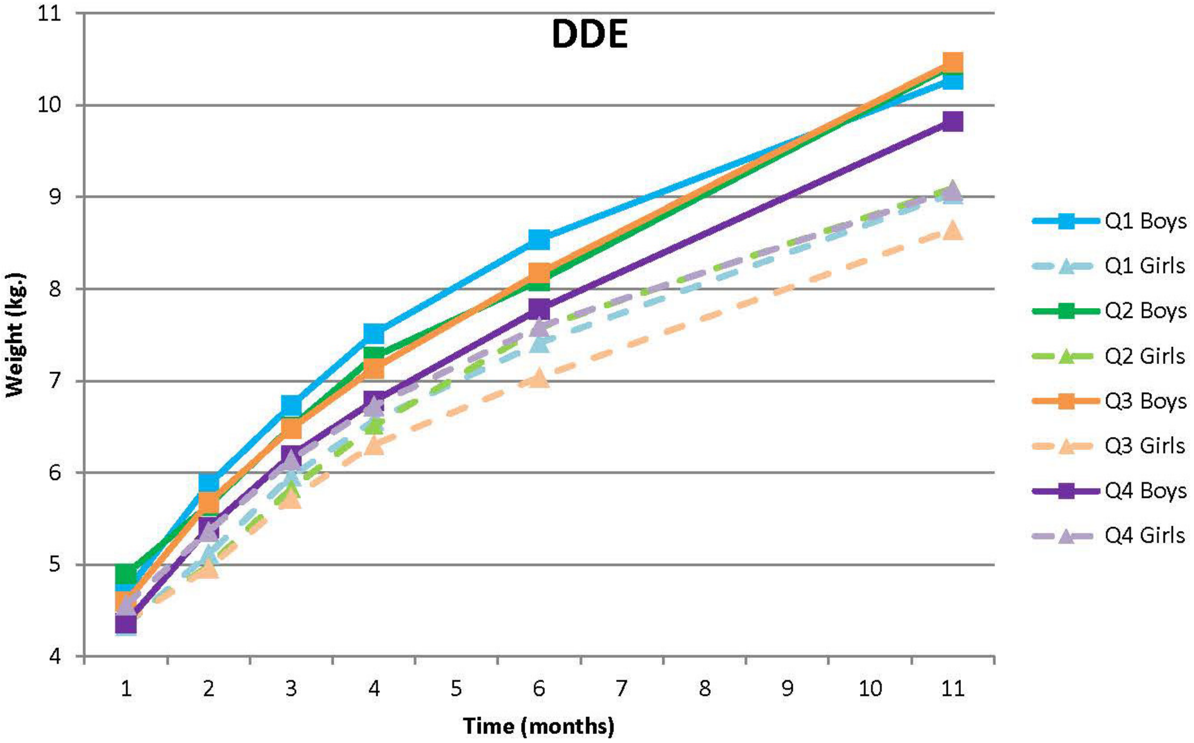

(**A**)

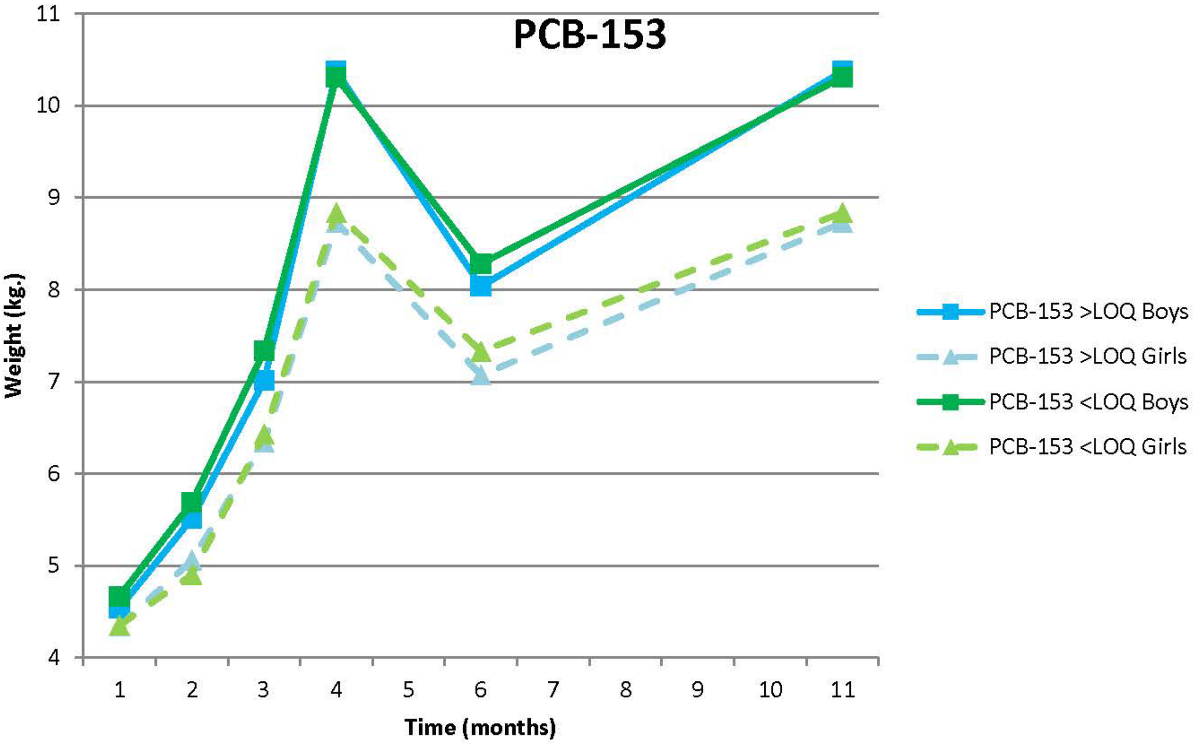

(**B**)

Figure S2. *Cont.*

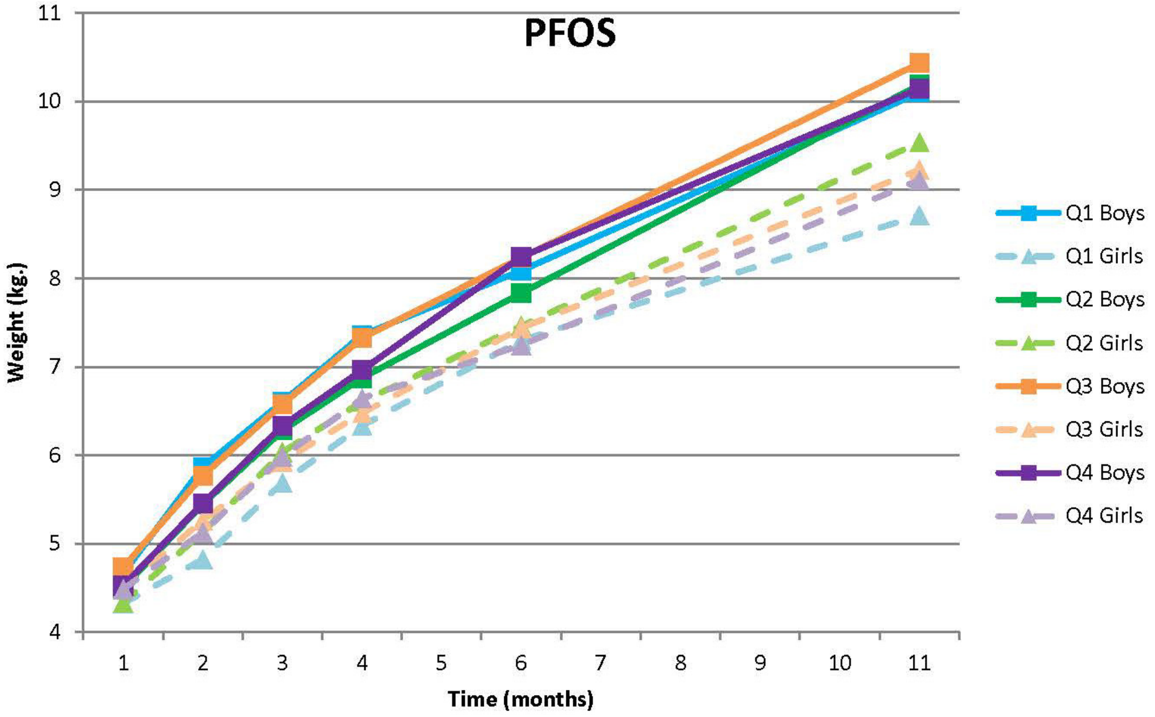

(**C**)

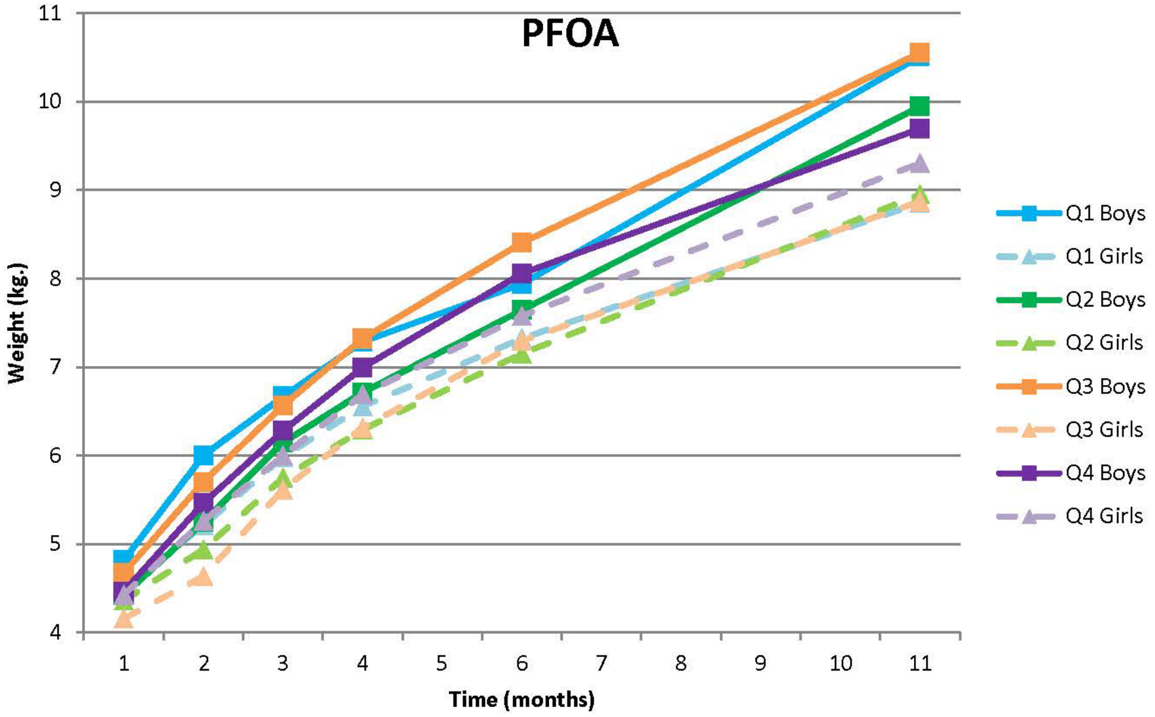

(**D**)

Figure S2. *Cont.*

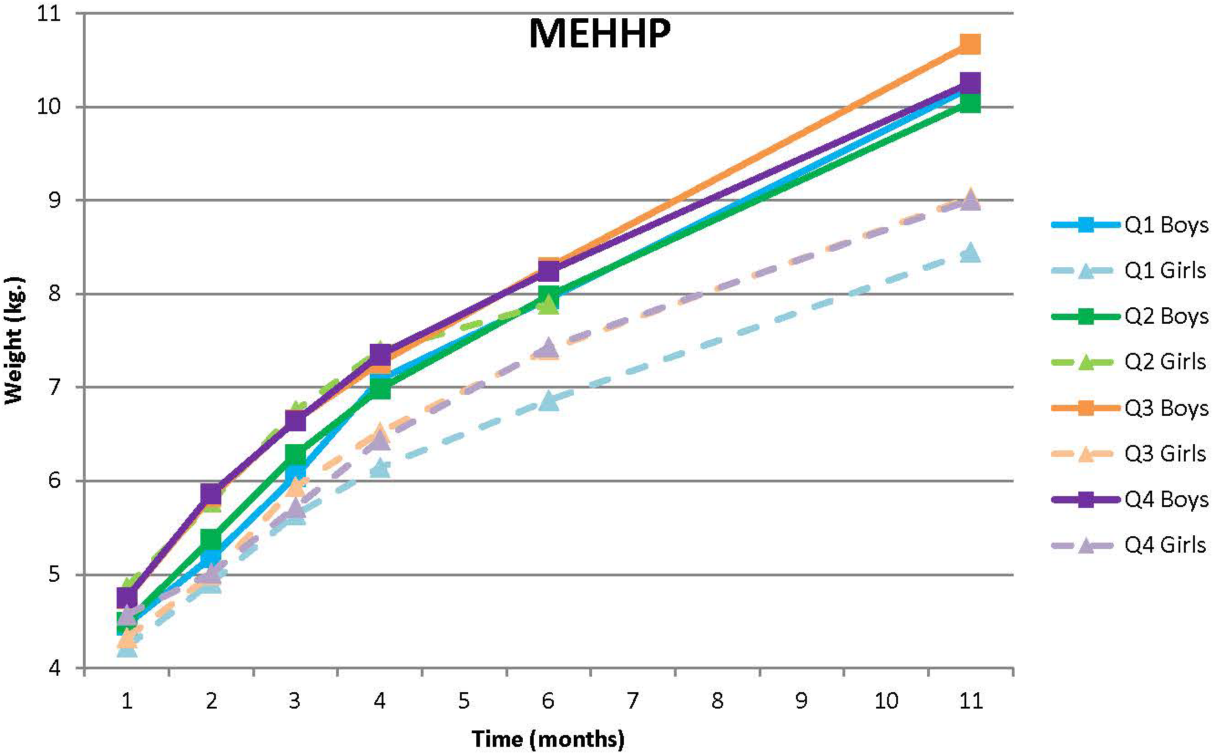

(**E**)

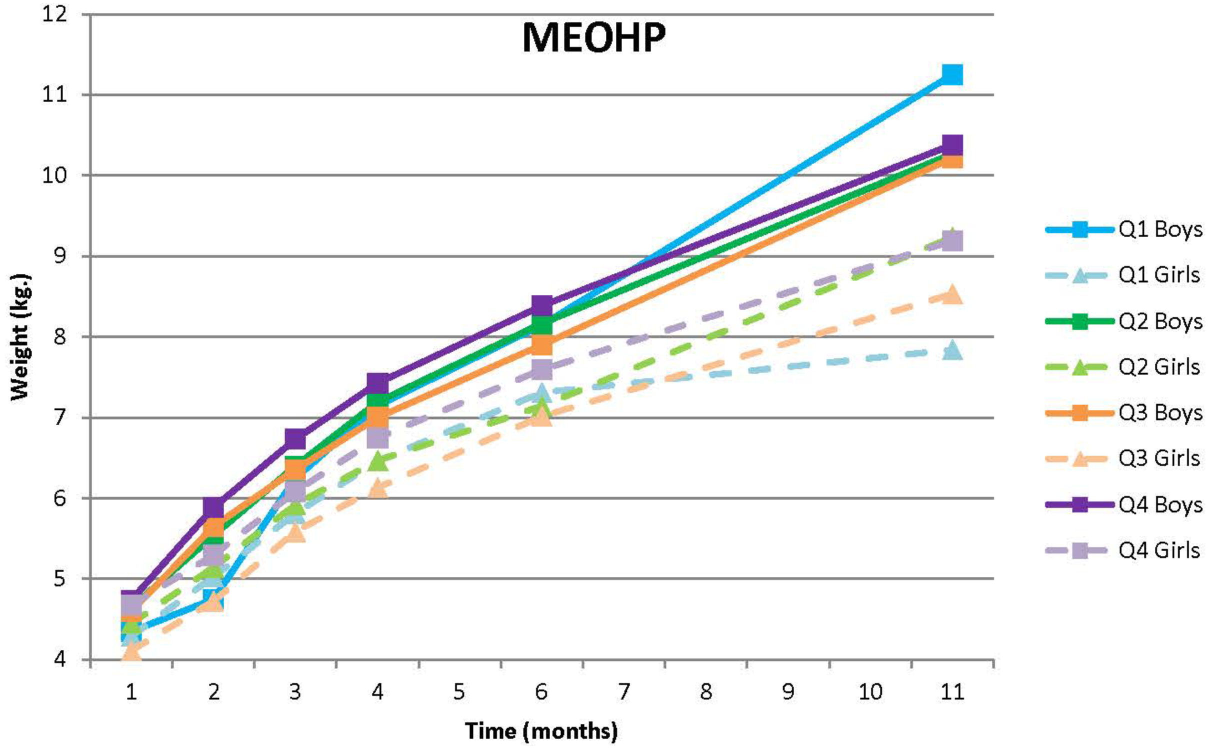

(**F**)

Figure S2. *Cont.*

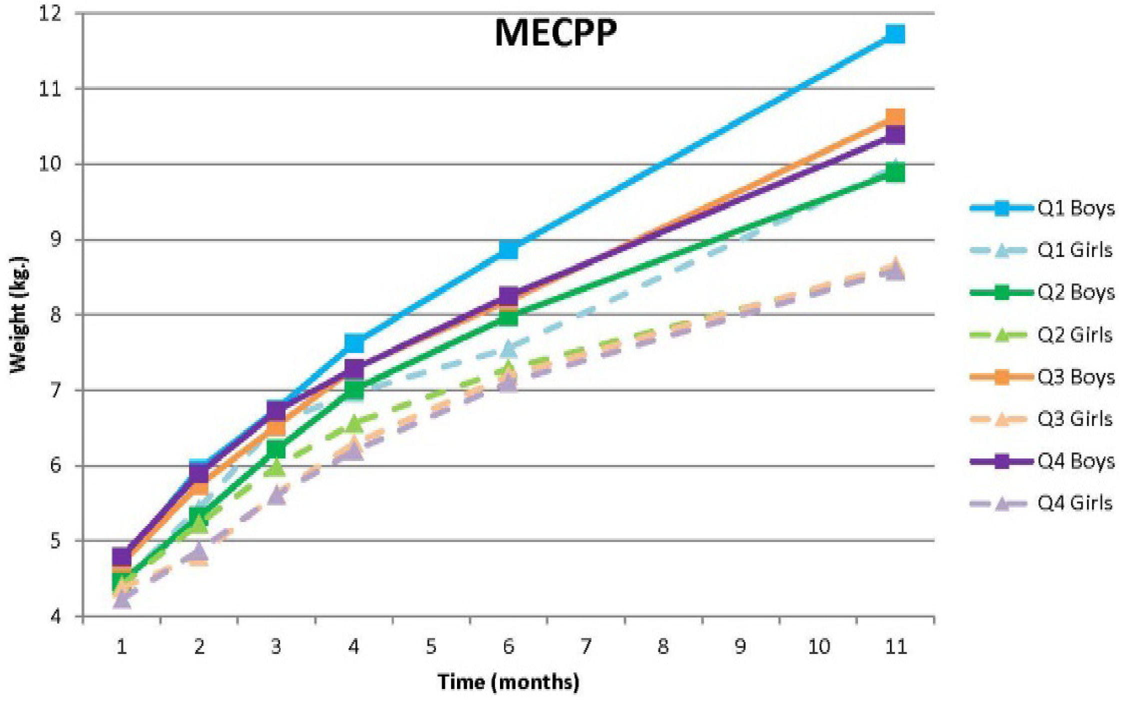

(**G**)

Figure S3. Sex specific height curves for early life DDE (**A**), PCB-153 (**B**), PFOS (**C**), PFOA (**D**), MEHHP (**E**), MEOHP (**F**) and MECPP (**G**) exposure.

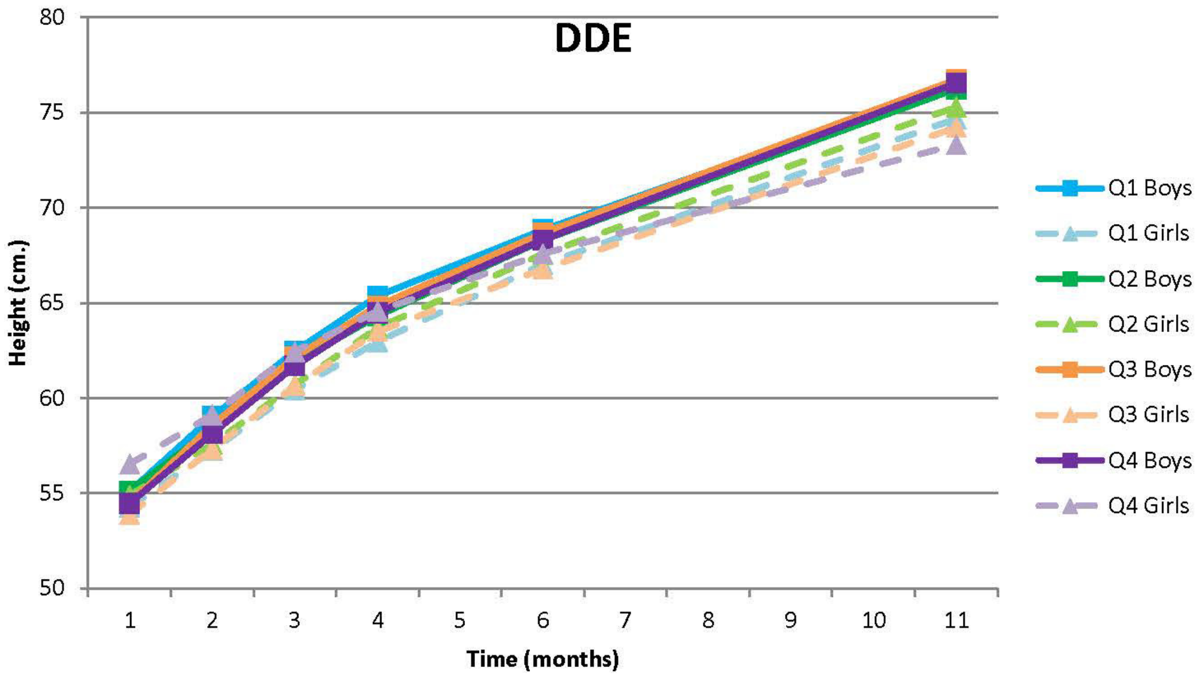

(**A**)

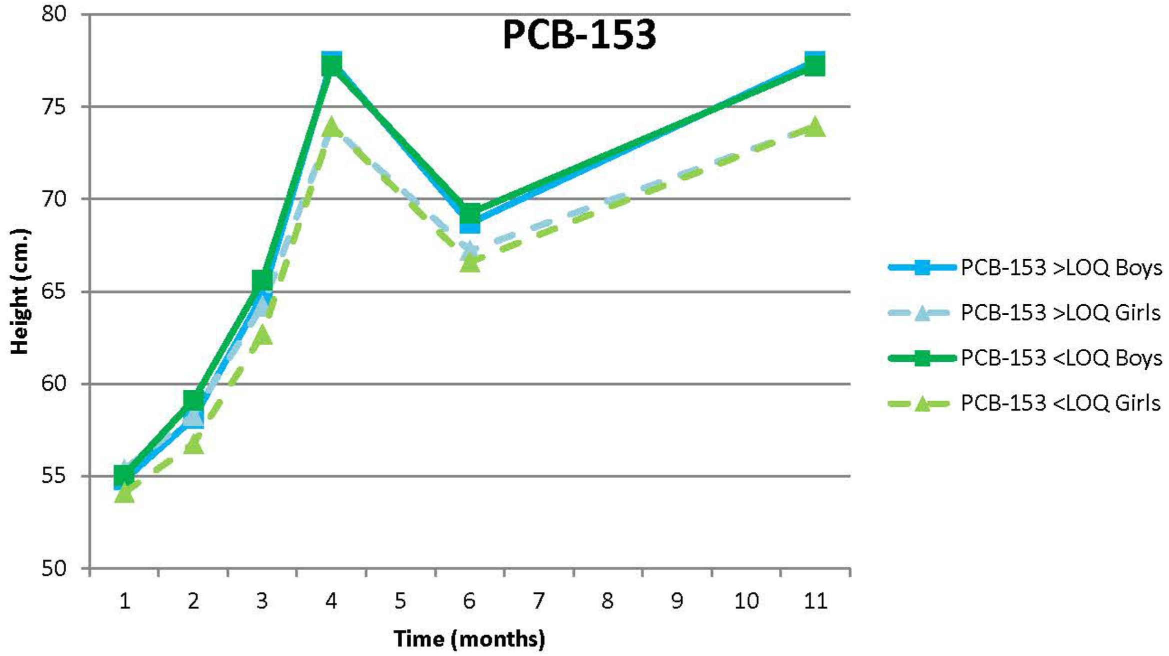

(**B**)

Figure S3. *Cont.*

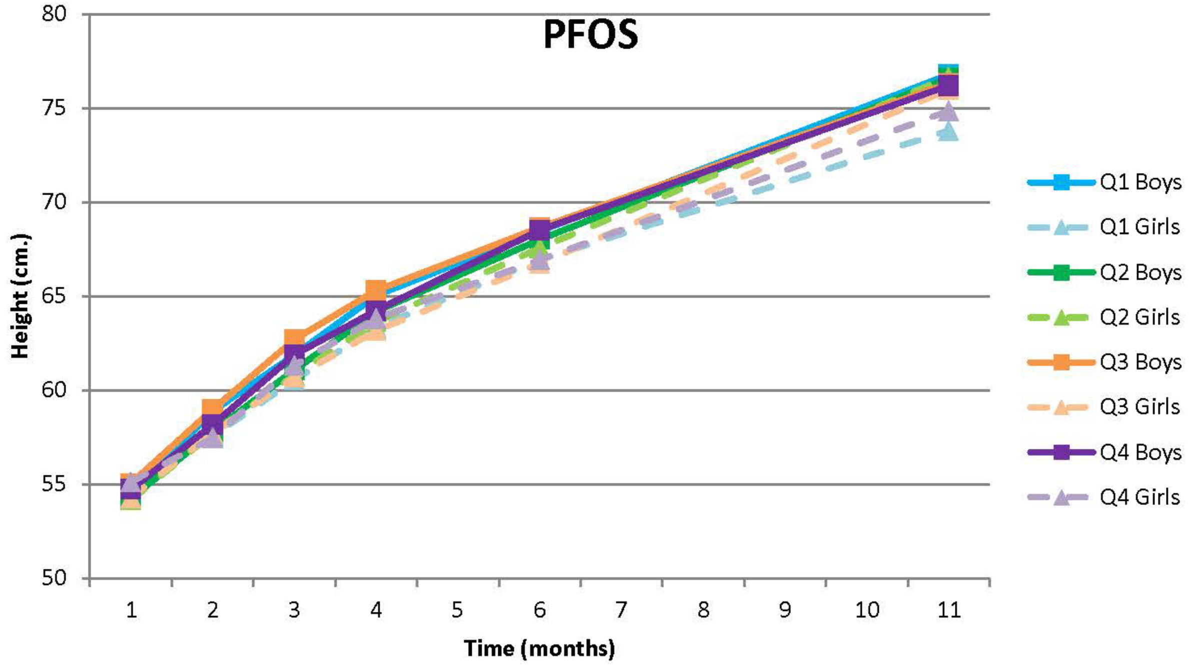

(**C**)

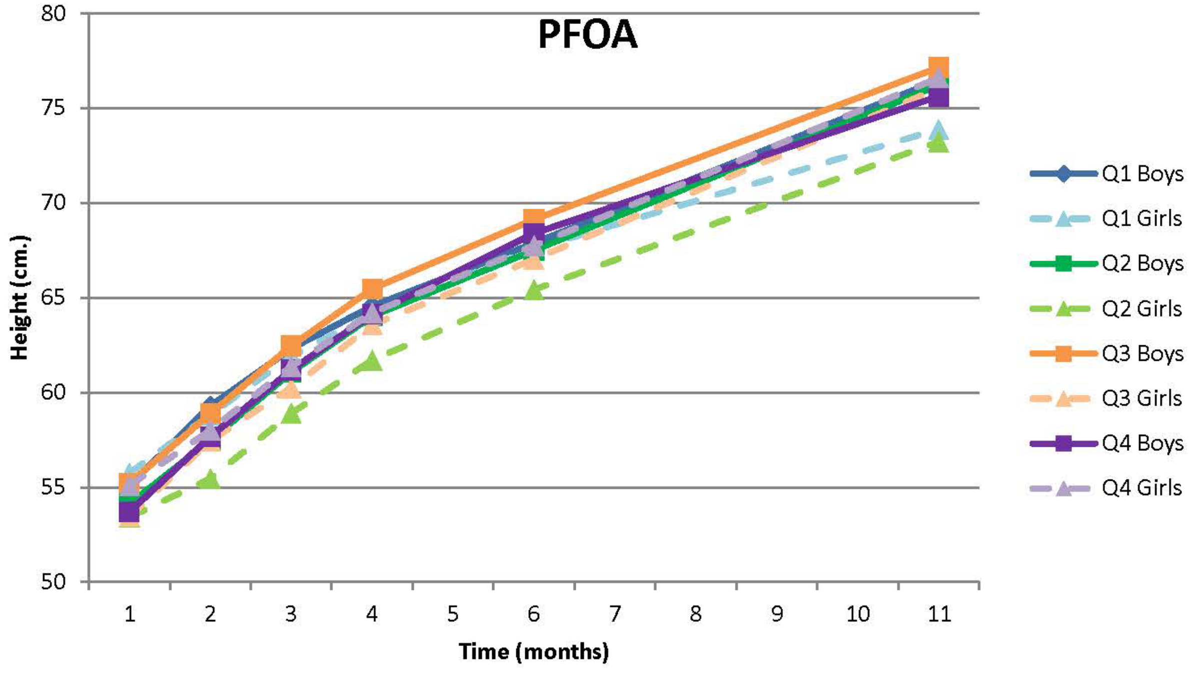

(**D**)

Figure S3. *Cont.*

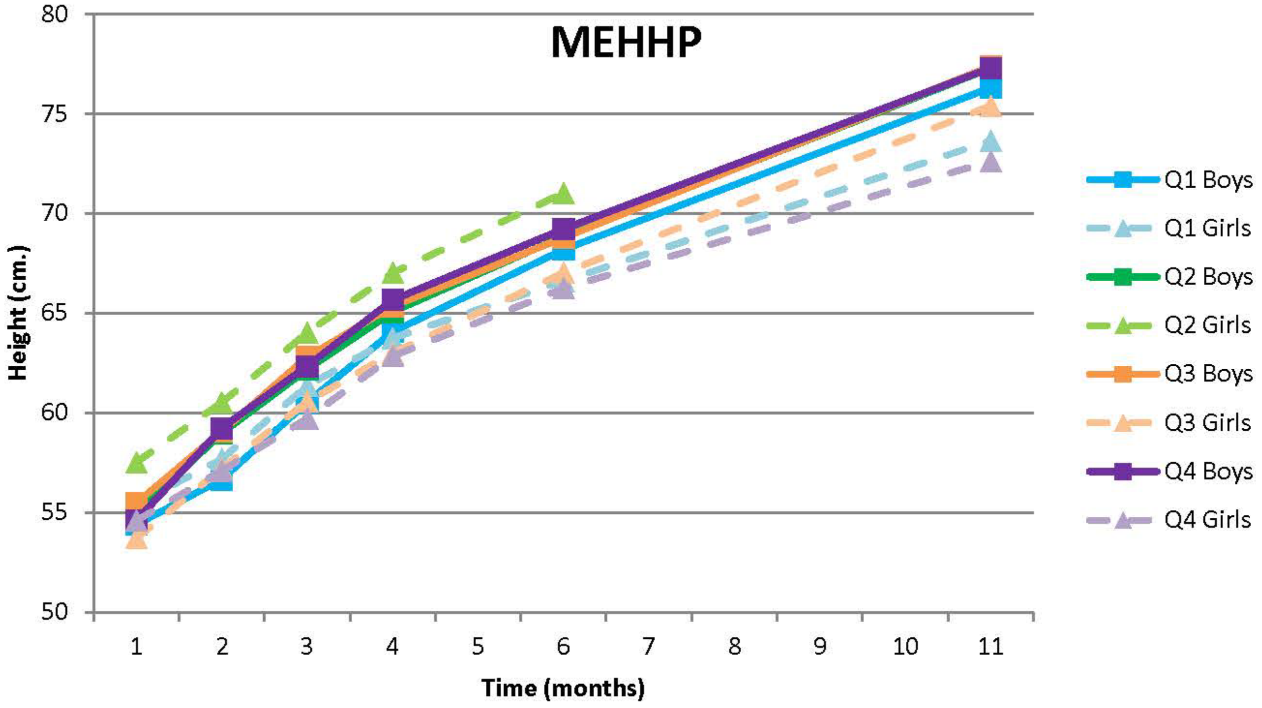

(**E**)

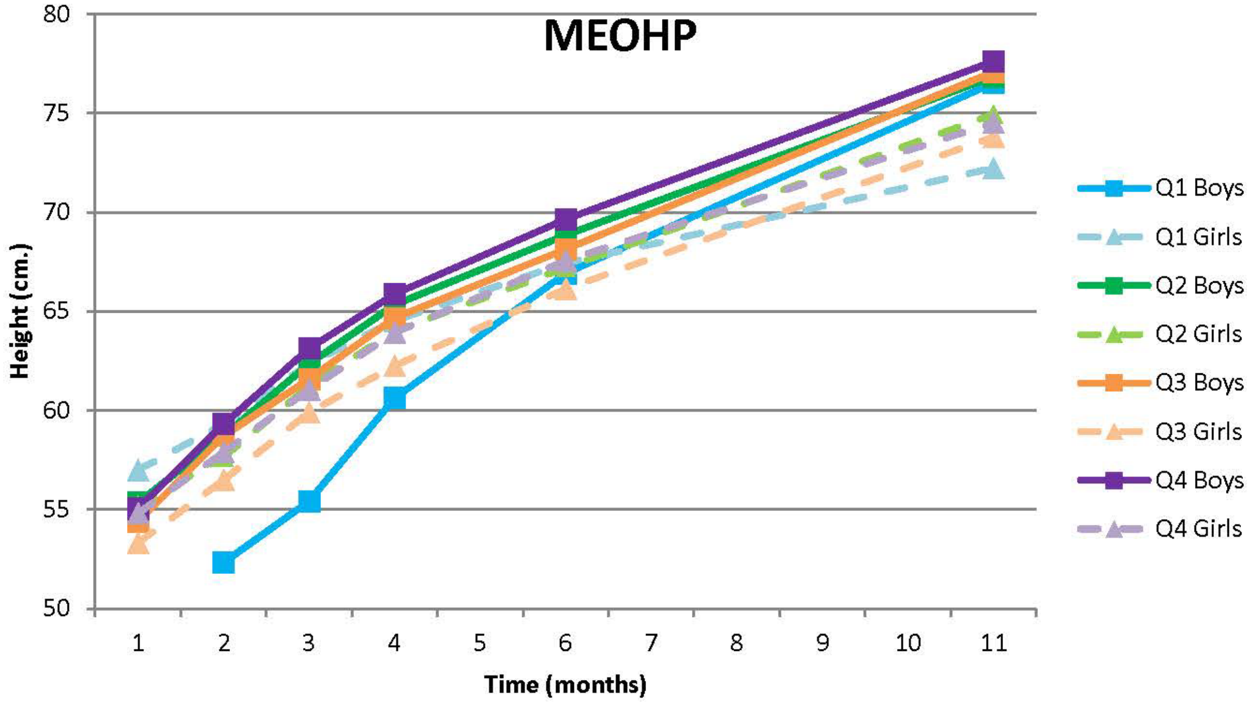

(**F**)

Figure S3. *Cont.*

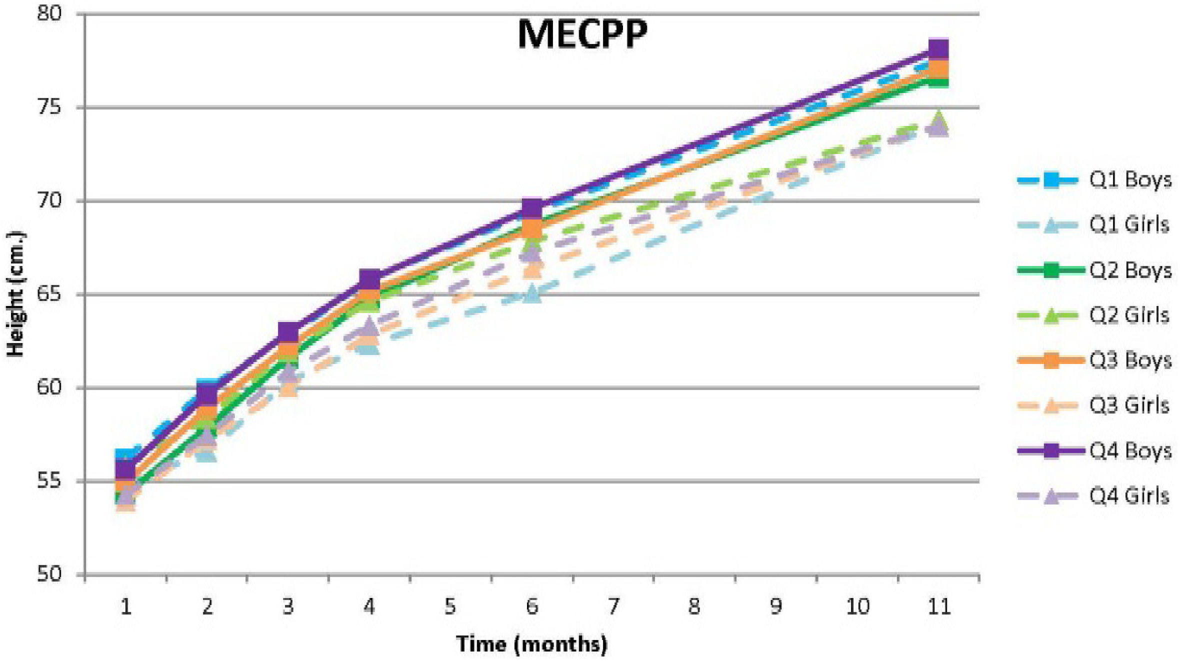

(**G**)

Figure S4. Sex specific head circumference curves for early life PFOS (**A**), PFOA (**B**) and PCB-153 (**C**) exposure.

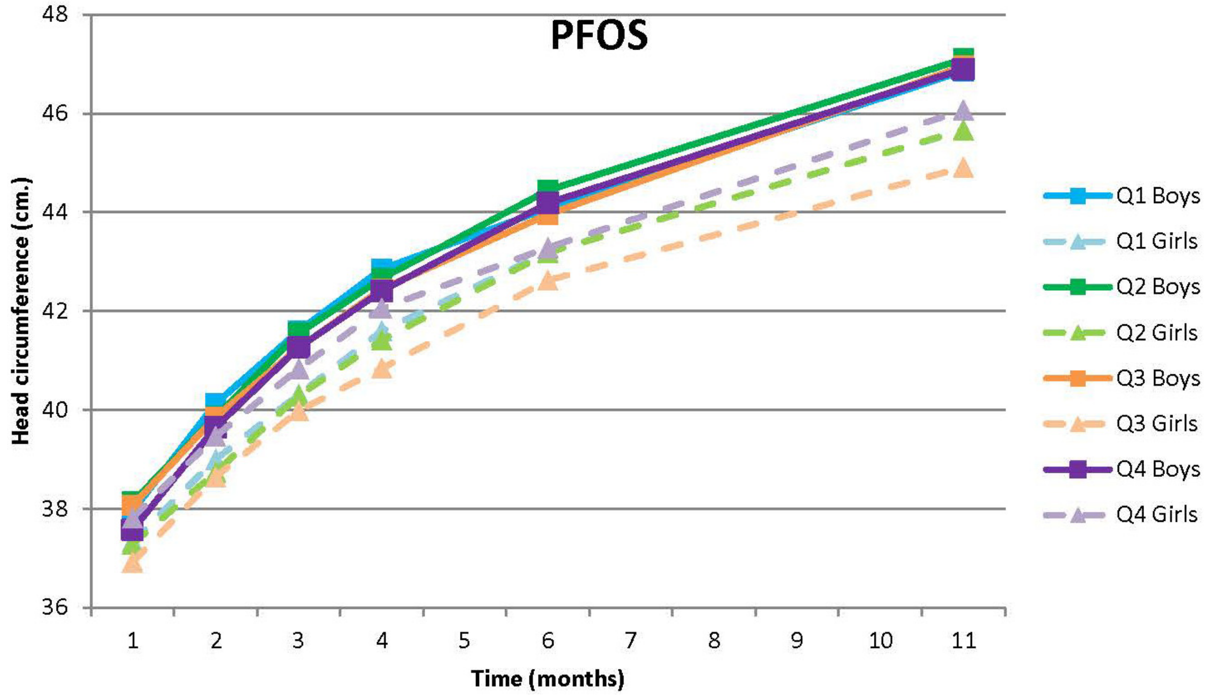

(**A**)

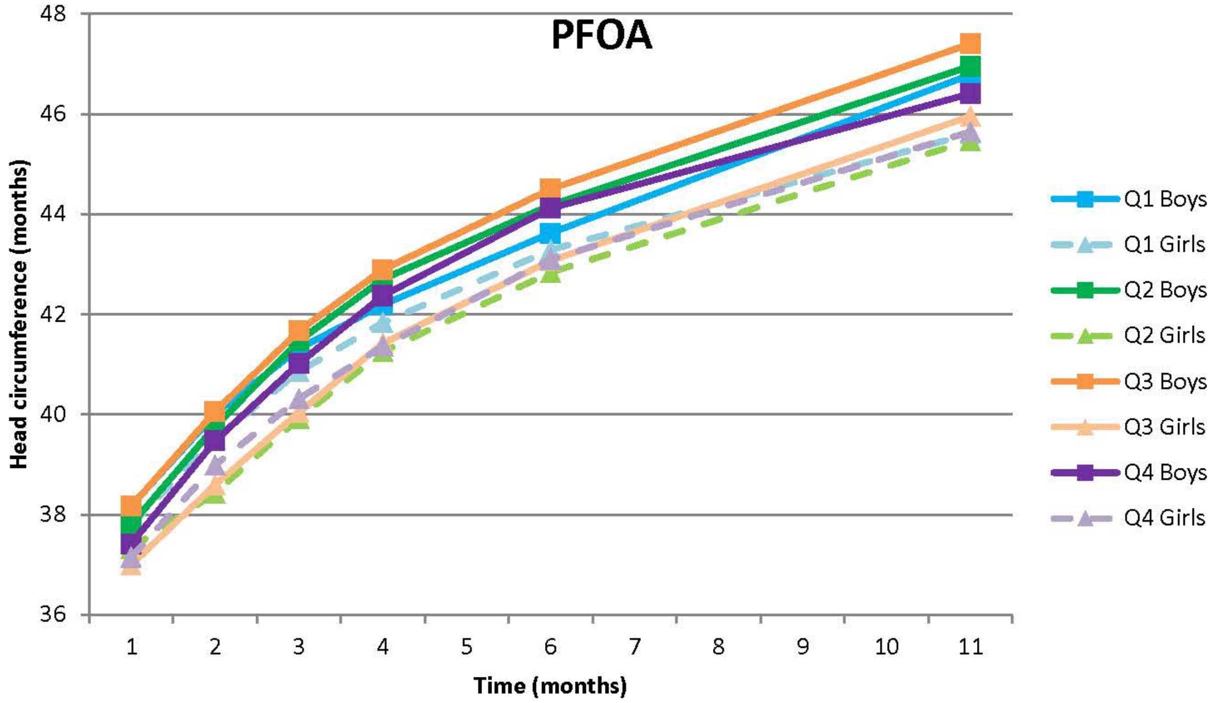

(**B**)

Figure S4. *Cont.*

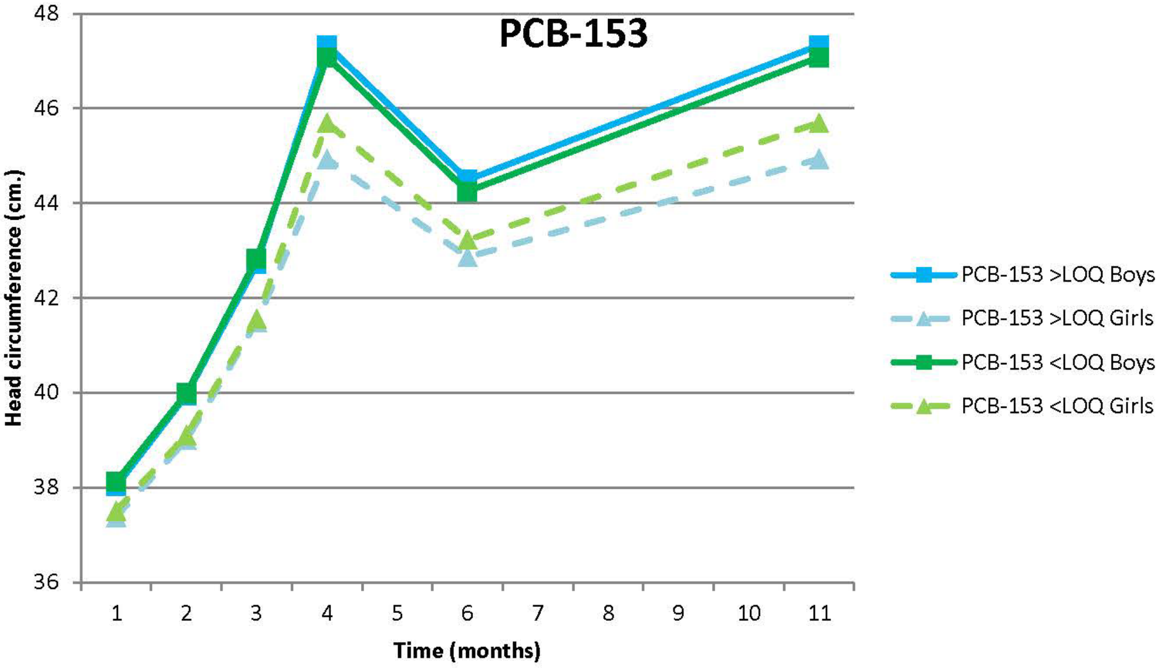

(**C**)

Reference

1. Manirakiza, P.; Covaci, A.; Schepens, P. Comparative Study on Total Lipid Determination using Soxhlet, Roese-Gottlieb, Bligh & Dyer, and Modified Bligh & Dyer Extraction Methods. *J. Food Compos. Anal.* **2001**, *14*, 93–100.

© 2014 by the authors; licensee MDPI, Basel, Switzerland. This article is an open access article distributed under the terms and conditions of the Creative Commons Attribution license (http://creativecommons.org/licenses/by/3.0/).
